# Voltage collapse in complex power grids

**DOI:** 10.1038/ncomms10790

**Published:** 2016-02-18

**Authors:** John W. Simpson-Porco, Florian Dörfler, Francesco Bullo

**Affiliations:** 1 Department of Electrical and Computer Engineering, Engineering Building 5, University of Waterloo, Waterloo, Ontario, Canada N2L 3G1; 2 Automatic Control Laboratory, Swiss Federal Institute of Technology (ETH), Physikstrasse 3, CH-8092 Zürich, Switzerland; 3 Department of Mechanical Engineering, Center for Control, Dynamical Systems and Computation, Engineering Building II, University of California at Santa Barbara, Santa Barbara, 93106-9560 California, USA

## Abstract

A large-scale power grid's ability to transfer energy from producers to consumers is constrained by both the network structure and the nonlinear physics of power flow. Violations of these constraints have been observed to result in voltage collapse blackouts, where nodal voltages slowly decline before precipitously falling. However, methods to test for voltage collapse are dominantly simulation-based, offering little theoretical insight into how grid structure influences stability margins. For a simplified power flow model, here we derive a closed-form condition under which a power network is safe from voltage collapse. The condition combines the complex structure of the network with the reactive power demands of loads to produce a node-by-node measure of grid stress, a prediction of the largest nodal voltage deviation, and an estimate of the distance to collapse. We extensively test our predictions on large-scale systems, highlighting how our condition can be leveraged to increase grid stability margins.

## Abstract (web summary)

A power grid is constrained by both its nonlinear physics and network structure, and violations of these constraints may lead to voltage collapse blackouts, which have been studied mostly numerically. Here the authors derive a closed-form condition to provide an analytic test for voltage collapse.

## Graphical abstract

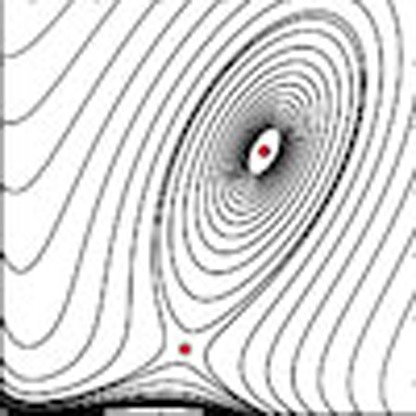

Modern power grids are some of the largest and most complex engineered systems. Currently however, growing consumer demand and the transition to distributed and deregulated small-scale generation are leading to increased system stress, and grid operators have strong economic incentives to operate networks close to their physical limits^1^,^2^,^3^. When these physical limits are approached or breached, power systems can experience a form of network-wide failure termed voltage collapse^4^,^5^,^6^,^7^,^8^. Voltage collapse and related instabilities have been identified as contributing factors in several recent large-scale blackouts, including Scandinavia (2003), the northeastern United States (2003), Athens (2004) and Brazil (2009) (refs 7, 8, 9). An obstacle in predicting voltage collapse is the extensive use of capacitor banks to hold up voltage levels at substations and along transmission lines. This voltage support keeps the system within operational constraints, but conceals the low stability margin of the network, leading to increased blackout risk^7^,^10^. Voltage fluctuations are presently being further aggravated by the increasing integration of utility-scale wind and photovoltaic sources. A key problem is therefore to develop physically insightful, easily computable stability conditions under which a network is safe from voltage collapse.

Applications of network theory and statistical mechanics to power transmission networks have to this point focused heavily on synchronization^11^,^12^,^13^,^14^,^15^,^16^,^17^,^18^,^19^, a phenomenon associated with the self-stabilizing collective behaviour of synchronous generators^20^. Synchronization is primarily controlled by the flow of active power; the real power used by loads to do work^8^. Interest in synchronization has led to a robust theoretical understanding of active power^1^,^16^,^21^,^22^,^23^, and a plethora of closed-form conditions under which power networks synchronize. In contrast, voltage collapse—a collective nonlinear ‘instability'^4^,^5^,^7^—has received little attention from a network perspective.

While voltage collapse is a multifaceted phenomena involving generator and transformer limits, the most important fundamental effect is a saddle-node bifurcation of the network equations, resulting in the loss of system equilibrium. Voltage phenomena are driven primarily by ‘reactive power', a much less intuitive concept than active power. Reactive power represents the ebb and flow of energy in the electromagnetic fields of system components. This energy is stored and released during each a.c. cycle, allowing system components to function normally and to facilitate the transfer of useful active power with minimal transmission losses^7^. Understanding and controlling reactive power is therefore essential for the efficient and safe operation of the grid.

Theoretical understanding of reactive power flow and voltage collapse in complex networks is poor, however, and numerical simulation is currently the only satisfactory approach to guard against voltage collapse; see refs 4, 5, 7, 24, 25, 26, 27, 28, 29 for numerical tests based on sensitivity matrices, and refs 1, 10, 23, 30, 31, 32 for approaches based on continuation methods, optimization and energy methods. The network is usually analysed not only under normal conditions, but under a large set of contingencies generated from single-component failures. A broad survey of computational approaches can be found in ref. 33. While effective computational tools in practice, these numerical approaches often offer little theoretical insight into how the underlying parameters and network structure influence voltage stability. An exception is the branch flow monitoring approach in refs 34, 35, where voltage collapse and network structure are linked by showing that collapse is preceded by the saturation of transfer paths between sources and sinks of power (Supplementary Note 2).

In contrast with computational methods focused on predicting voltage collapse with great accuracy, here we develop a simple and new analytical framework for analysing voltage collapse, and focus in particular on understanding how the structure of the network influences stability margins. While previous analytic works^36^,^37^ have relied on spectral graph measures such as algebraic connectivity^13^,^14^,^16^, the closed-form voltage stability condition, we propose below accounts for the grid structure by simultaneously incorporating all eigenvalues of an appropriate system matrix, and combines this information with the sizes and locations of shunt capacitors and loads. To our knowledge, this stability condition is the first to achieve this combination. Our analysis, which is based on a simplified power flow model, yields predictions for the voltage profiles of power grids and provides an explicit stability margin against voltage collapse. The predictions are found to be quite accurate in standard test cases. Our approach is not only mathematically accurate, but also appealing and intuitive to scholars versed in network science and dynamic processes over networks. Since we focus on the influence of grid structure on voltage collapse, we analyse the simplest possible network model that captures the essential bifurcation phenomena; we discuss important extensions involving second-order effects due to active power coupling, as well as component failures in the ‘Discussion' section. While our simplified model does not account for active power coupling, we show through extensive numerical experiments that our predictions remain robust when including these effects, and we specifically highlight when they break down.

## Results

### Power network modelling

We consider a high-voltage power network with *n*≥1 load nodes and *m*≥1 generator nodes, and in this article we focus on the decoupled reactive power flow equations


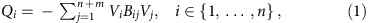


where *Q*_*i*_ (resp. *V*_*i*_) is the reactive power demanded (resp. voltage magnitude) at load *i*∈{1,…,*n*}. Voltage magnitudes *V*_*j*_ at generators nodes *j*∈{*n*+1,…,*n*+*m*} are regulated by internal controllers to constant values, and the sum in equation (1) therefore contains both quadratic and linear terms in the unknown load voltages **V**_L_=(*V*_1_,…,*V*_*n*_). The symmetric coefficients *B*_*ij*_=*B*_*ji*_ quantify the effective strength of connection between nodes *i* and *j*. These coupling coefficients have the form *B*_*ij*_=*b*_*ij*_ cos(*θ*_*i*_−*θ*_*j*_), where *b*_*ij*_≥0 quantifies the strength of the transmission line joining nodes *i* and *j*, and *θ*_*i*_−*θ*_*j*_ is the difference between the angles of the voltage phasors at the two nodes. These phase angles may be estimated in advance using a decoupled active power flow model^38^, or come from the output of a numerical power flow solver. The diagonal elements are defined by *B*_*ii*_=−∑_*j*≠*i*_ *b*_*ij*_+*b*_*ii*_, where *b*_*ii*_ accounts for inductive or capacitive shunts (connections to ground). The sparsity pattern of the matrix *B*_*ij*_ therefore encodes both the structure of the physical network and the degree of coupling between nodes after accounting for active power transfers. Equation (1) arises from considering the balance of reactive power at each node in the network while neglecting second-order effects accounting for coupling with active power flows and phase-angle dynamics; more modelling information may be found in (Supplementary Note 3).

**Figure 1:**
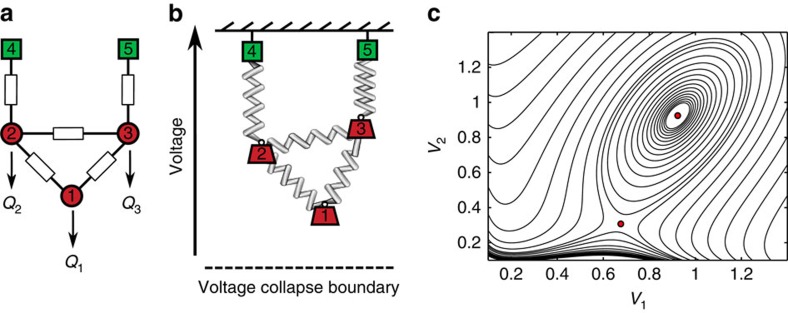
Mechanical and energy interpretations of power flow. (**a**) An example power network with two generators (green) supplying power to three loads (red). Power demands (*Q*_1_, *Q*_2_, *Q*_3_) are placed on the load nodes; (**b**) a mechanical analogy: a linear spring network placed in a potential field. The generator voltages (green) are ‘pinned' at constant values, while the load voltages (red) are masses ‘hanging' off the generators, their equilibrium values being determined by their weights (the power demands **Q**_L_=(*Q*_1_, *Q*_2_, *Q*_3_)), the heights of the fixed-generator voltages (*V*_4_, *V*_5_), and by the stiffness of the spring network (the susceptance matrix **B**). Voltage collapse can occur when one of the masses crosses an appropriate collapse boundary curve; (**c**) Contour plot of energy function when *Q*_3_=0 and node 3 is eliminated via Kron reduction^13^. Since *E*(**V**_L_) contains logarithms, it tends to −∞ as either axis is approached. In a normalized system of units, the stable high-voltage equilibrium rests in a local minimum at (0.94, 0.94), while an unstable low-voltage equilibrium sits at the saddle (0.68, 0.30). Voltage collapse occurs when these equilibria coalesce and the system trajectory diverges.

A novel mechanical analogy for the power flow (1) is shown in Fig. 1b. The equilibrium configuration of the spring network corresponds to the desirable high-voltage solution of (1), and can be interpreted as a local minimum (Fig. 1c) of the energy function^31^


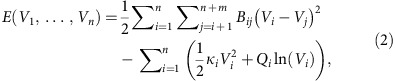


where 
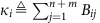
 (Supplementary Note 4). Note that the power demands *Q*_*i*_ generate a logarithmic potential, leading to multiple equilibria (Fig. 1c). Standard practice is that for stable and economical network operation with minimal transmission losses, nodal voltages should remain near their open-circuit values as obtained for an unloaded (and thus unstressed) network^8^. Intuitively then, a stable steady-state is characterized by


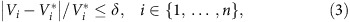


where 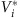 is the open-circuit voltage at the *i*th node and *δ*>0 is a dimensionless variable quantifying an allowable percentage limit on deviations. Intuition from Fig. 1 suggests that a stiff, lightly loaded grid will have a high and uniform voltage profile with small deviation *δ*, while a weak, heavily loaded grid will result in voltage collapse. The following ‘Analytic results' section will make this intuition precise and mathematically accurate.

### Analytic results

We suggest that for assessing voltage stability and collapse, one should consider not the underlying electrical network encoded in the susceptance matrix **B**, but a reduced and re-weighted auxiliary network. This auxiliary network shares the same topology as the physical network, but with new edge weights which encode both generator voltage levels and the topology and strength of connections between loads and generators. After potentially reordering the network nodes so that loads and generators are labelled, respectively, {1,…,*n*} and {*n*+1,…,*n*+*m*}, we may partition the (*n*+*m*) × (*n*+*m*) coupling matrix **B** with elements *B*_*ij*_ into four block matrices as


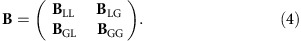


The *n* × *n* sub-matrix **B**_LL_ now describes the interconnections among loads, while the *n* × *m* matrix **B**_LG_ specifies the interconnections between loads and generators. This partitioning suggests a natural mapping from generators to loads through the matrix 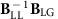, which we can use to define the open-circuit load voltages 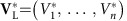 by


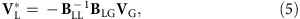


where **V**_G_=(*V*_*n*+1_,…,*V*_*n*+*m*_) is the vector of fixed-generator voltages. To quantify the stiffness of the spring network in Fig. 1b, we combine the nominal voltages in equation (5) with the sub-matrix **B**_LL_ in equation (4) to obtain the symmetric stiffness matrix


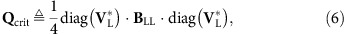


where 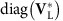 is the matrix with 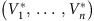 on the main diagonal. In other words, **Q**_crit_ has units of power and its *ij*th entry is given by 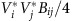. Selected topological features, edge weights, generator voltages, and the relative locations of generators and loads are all concisely encoded in the stiffness matrix **Q**_crit_.

Just as the stiffness matrix of a standard spring network relates displacements to spring forces, the matrix **Q**_crit_ can be thought of as relating the dimensionless voltage deviations 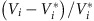 to the reactive power demands **Q**_L_=(*Q*_1_,…,*Q*_*n*_). Indeed, this normalization to dimensionless variables is key to our theoretical analysis. To arrive at small normalized deviations of the form (3), it then seems reasonable that the dimensionless matrix-vector product 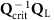 should be small in some sense. Our main result below shows that this intuition based on linear spring networks can be made precise, leading to guarantees on voltage deviations for the nonlinear network (1). A derivation and a formal proof can be found in the ‘Methods' section and in Supplementary Note 5 respectively.

*Theorem 1*: The power flow equations (1) have a unique, stable, high-voltage solution (*V*_1_,…,*V*_*n*_) if


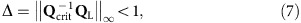


where 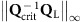 is the largest magnitude of the entries of the vector 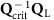. Moreover, each component V_*i*_ of the unique high-voltage solution satisfies the bound 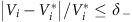, where 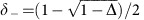.

The matrix-vector product 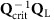 captures the interaction between the auxiliary network structure and the locations of loads, with the infinity norm 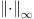 identifying the maximally stressed node. The scalar *δ*_−_ then bounds the largest voltage deviation in the network. No reactive loading corresponds to zero stress Δ=0 and *δ*_−_=0; voltages align with their open-circuit values. Conversely, when Δ=1, the network's guaranteed stability margin has been depleted. Said differently, Δ<1 guarantees the existence of a stable equilibrium, while Δ≥1 is a necessary condition for voltage collapse, where at least one node of the network has become overly stressed. The stability condition (7) can be therefore be interpreted as a dual to previous literature showing that voltage collapse is always preceded by at least one edge of the network becoming overly stressed^34^,^35^. Moreover, the bound Δ<1 is the ‘tightest' possible general bound, as cases can be constructed where voltage collapse occurs at Δ=1 (Supplementary Note 5). Note that equation (7) captures the desired intuition of the spring network analogy in Fig. 1b; the network stiffness matrix **Q**_crit_ should be large when compared with the reactive loading **Q**_L_; see (Supplementary Note 5) for complex network, power system and circuit-theoretic interpretations of the stability condition. In terms of Fig. 1c, 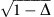 lower bounds the distance in voltage-space between the stable and unstable equilibria in the power system energy landscape. In summary, the stability condition (7) concisely and elegantly captures the physical intuition developed in Fig. 1 and in the previous section, and guarantees the existence of a unique equilibrium for the nonlinear network equation (1).

For fixed reactive demands **Q**_L_, the stability test (7) states that the largest stability margins are obtained by making 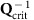 small. Since the parameters of the grid are embedded in the stiffness matrix **Q**_crit_ defined in equation (6), the stability test (7) provides insight into how the parameters of the network influence its stability margins. Rigorous statements may be found in Supplementary Note 6, while here we present the key insights. For example, by examining the definitions (5) and (6) one observes that raising generator voltage levels **V**_G_ will weaken (in magnitude) the elements of 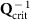 and therefore increase stability margins. In terms of Fig. 1b, this corresponds to ‘raising the ceiling', which increases the distance to the stability boundary. Since the coupling weights *B*_*ij*_ enter the stiffness matrix (6) both directly and through the open-circuit voltages 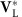, their effects on stability margins are subtle, and counter-examples can be constructed where increasing the coupling between generators and loads decreases stability margins (Supplementary Note 6). Nonetheless, one may show rigorously that under normal network conditions, strengthening the edge weights *B*_*ij*_ between loads and generators and increasing the shunt capacitances *b*_*ii*_ at loads are both beneficial to stability margins. The first corresponds to stiffening the springs (4, 2) and (5, 3) of Fig. 1b, while the second can be thought of as extra upward force directly applied to nodes {1, 2, 3}. In summary, the stability condition (7) can be leveraged to provide new qualitative insights into how the network structure and parameters influence stability margins.

Finally, in contrast to standard voltage collapse studies, note that we have made no assumptions about the direction of the reactive power demands **Q**_L_, which appear linearly in equation (7). Therefore, the condition (7) simultaneously accounts for all directions in the space of reactive power demands. This generality may result in the test (7) being conservative for a particular direction in the space of power demands. On the other hand, this generality allows one to assess network stability for an entire set of possible power demands via a single evaluation of the condition (7).

**Figure 2:**
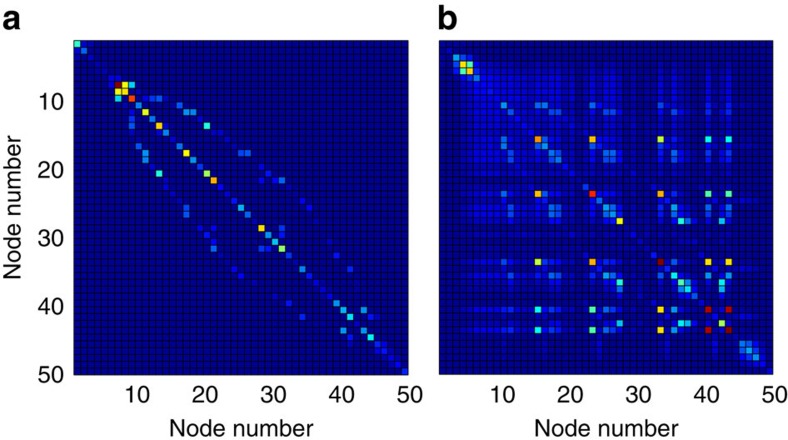
Sparsity patterns of network matrices for 57 node test case. (**a**) the stiffness matrix **Q**_crit_ representing the auxiliary network. (**b**) The inverse stiffness matrix 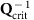. The 57 node network contains 50 loads and 7 generators. Nodes are sorted and grouped by connected components of the subgraph induced by **Q**_crit_, with connected components ordered from largest to smallest; nodes {1,…,48} are part of one large connected component, while nodes {49, 50} each constitute their own component. Colour scale represents normalized values of the matrix elements, with dark blue being zero and red being one. Diagonal elements of **Q**_crit_ are displayed in absolute value for clarity.

The inverse of the stiffness matrix is the sensitivity matrix relating percentage changes in voltage to changes in reactive demands **Q**_L_, as can be seen from the linearized relationship 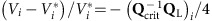. A comparison of the stiffness matrix **Q**_crit_ and its inverse is shown in Fig. 2. The stiffness matrix **Q**_crit_ is itself very sparse, mirroring the physical topology of the grid. This sparsity allows the inequality (7) to be rapidly checked by solving a sparse linear system **Q**_crit_**x**=**Q**_L_; the vector **x** serves as a linear approximation of (and an upper bound on) the exact voltage deviations 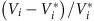. In contrast, the inverse 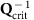 is a dense matrix with significant off-diagonal elements, indicating the importance of not only local but also multi-hop interactions. While we omit the details here, the stability condition (7) can be extended to additionally guarantee the satisfaction of hard, predefined limits on both voltage magnitudes and the reactive power injections of generators (Supplementary Note 5 and Supplementary Note 6, respectively).

### Numerical assessment of voltage stability condition

In this section we provide three numerical studies to assess the accuracy of the stability condition (7) in large-scale power networks, and to determine its predictive limitations. Our first study focuses on the accuracy of the theoretical bound 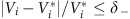 in typical networks operating in the normal regime far away from voltage collapse. We consider 11 widely established test cases^39^, ranging from a small 9 node network to a representation of the Polish grid with nearly 2,400 nodes. To generate a diverse set of sample networks, we construct 1,000 realizations of each network, with up to 30% deviation from forecast conditions in generation and up to 50% deviation in active and reactive power demands, drawn from a normal distribution centred around base conditions; see (Supplementary Methods) for details. For each realization, we solve the more-realistic lossless coupled active/reactive a.c. power flow equations numerically, and we compare the largest nodal voltage deviation 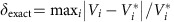 from the numerically determined voltage profile to the analytic bound 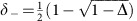 from our main result (7) based on the simplified model (1) with the numerically determined phase angles *θ*_*i*_−*θ*_*j*_ substituted.

Our findings are reported in Table 1. The theoretical prediction of the stability test (7) is that *δ*_exact_≤*δ*_−_; the first column indicates that this inequality held for all realizations for which the numerical solver converged. All realizations for which the numerical solver failed to converge were discarded; this occurred in fewer than 1% of all cases. The second and third columns list the average values of these two quantities over all realizations. As can be seen, the voltage deviations range from roughly 1% to 6% from open-circuit conditions. The final column shows the average of the prediction error (*δ*_−_−*δ*_exact_)/*δ*_exact_ over all realizations. For all networks from 9 to 2,383 nodes (except the 57 and 300 node networks) the prediction error is less than 1%, indicating that prediction accuracy is not directly dependent on system size. Perhaps surprisingly, considering the simplicity of the condition (7), the least accurate prediction overestimates voltage deviations by only 3.8%. We conclude that for normally stressed large-scale networks, the bounds predicted by the stability condition (7) hold and are accurate even when tested on more complicated coupled power flow models.

Our second study analyses the predictions of (7) in a highly stressed network, again for the more-realistic lossless coupled active/reactive power flow model. As our focus is on studying bifurcation phenomena for the network equations, we discard generator limitations in this study and assume internal generator controls hold the network-side generator voltages constant; see Supplementary Note 7 for theoretical extensions which include generator limits. As we noted previously, Δ≥1 is a necessary condition for voltage collapse, and we now test the gap between this necessary condition and true point of collapse. We consider the 39-node reduced representation of the New England power grid, illustrated in Fig. 5a. Beginning from normal base case loading conditions, the active and reactive power demands and generation are increased continuously along a chosen ray in parameter-space, with the size of the increase parameterized by a scalar *λ*, until voltage collapse occurred at a value *λ*=*λ*_collapse_. For each *λ*∈[0, *λ*_collapse_], we determine numerically the system equilibrium and recalculate Δ from equation (7) using the numerically determined phase angles *θ*_*i*_−*θ*_*j*_.

**Figure 3:**
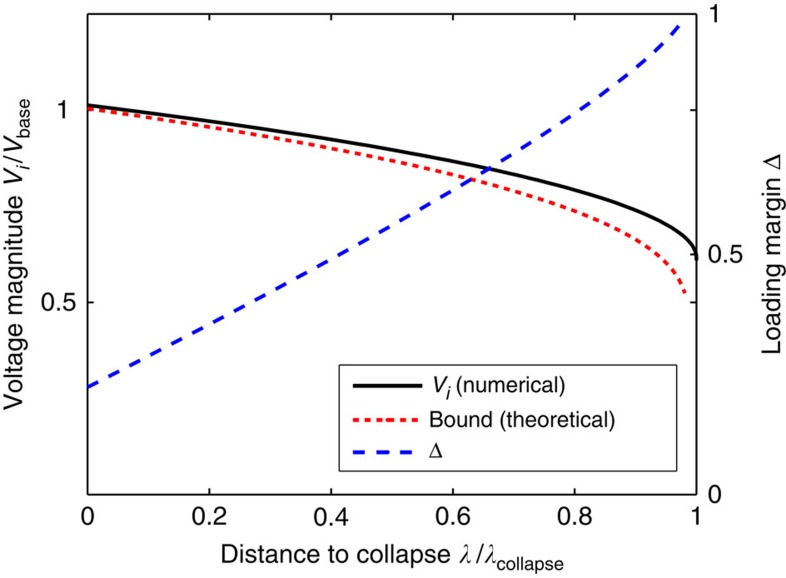
Stress testing of voltage stability condition for low power factor loading. The horizontal voltage axis is scaled by *V*_base_=345 kV. The solid black trace is the numerically computed voltage magnitude at node four, while the dotted red trace is given explicitly by 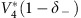, where *δ*_−_ is determined as below (7). The stability margin Δ is shown in dashed blue. When Δ>1, *δ*_−_ becomes undefined and the corresponding bound is no longer plotted.

The above testing procedure obviously depends on the choice of direction for increase in the space of power demands and generation. We select two directions and study them separately, to illustrate the strengths and limitations of our analytic approach based on a simplified power flow model. As a first choice, we select a direction where the mean power factor in the network is decreased 20% to a value of 0.7. (The power factor of the *i*th load is defined as 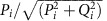, where *P*_*i*_ is the active power drawn by the load. If *P*_*i*_=*Q*_*i*_, then the power factor is 0.707.) This corresponds to a case where loads consume roughly equal amounts of active and reactive power, which in practice is unusually highly reactive power consumption. We therefore expect that instabilities associated with reactive power flow should dominate any unmodeled active power effects, and the simplified model (1) should serve as a good proxy for the coupled active/reactive power flow equations. As a function of *λ*, Fig. 3 displays the trace of the voltage magnitude at node 4 (solid black), the loading margin Δ (dashed blue), and the bound 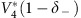 (dotted red) determined by equation (7). Node 4 was determined through equation (7) to be the most stressed node in the network, and hence the node for which our theoretical bound would be best tested. First, observe that the numerically determined voltage trace is bounded below by the trace of the theoretical bound, as expected. The loading margin Δ increases roughly linearly with *λ*, with Δ=1 occurring at *λ*/*λ*_collapse_=0.98. Our previous conclusions regarding the necessity of Δ>1 for voltage collapse therefore hold in this highly stressed case for the more complicated coupled active/reactive power flow model, and the gap between the necessary condition Δ>1 and the true point of collapse is a surprisingly small 2%.

**Figure 4:**
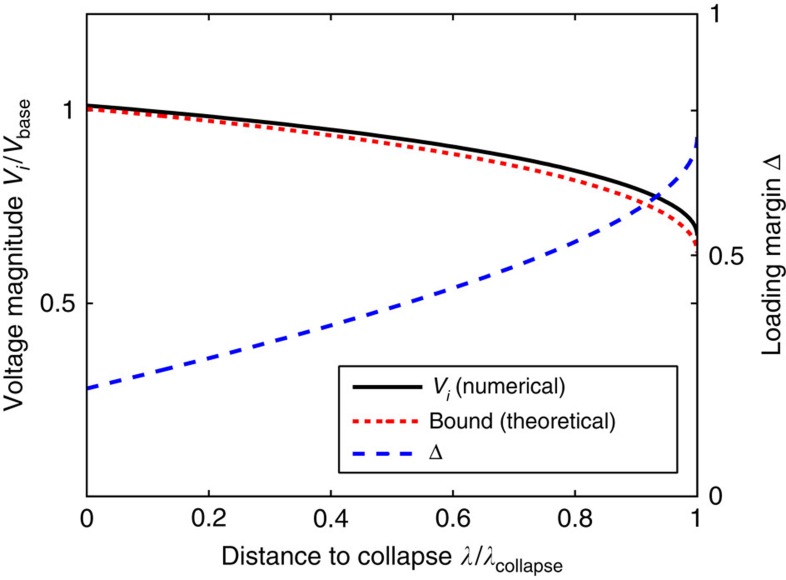
Stress testing of voltage stability condition for high power factor loading. The horizontal voltage axis is scaled by *V*_base_=345 kV. The solid black trace is the numerically computed voltage magnitude at node four, while the dotted red trace is given explicitly by 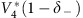, where *δ*_−_ is determined as below (7). The stability margin Δ is shown in dashed blue.

As a second loading direction for testing, we maintain the direction of the base case, for which the average power factor of loads is approximately 0.88. In this regime reactive power transfers will be less prominent, and we expect the unmodeled coupling between active and reactive power flows to induce voltage collapse at a loading level lower than expected from the simplified model (1). Again as a function of *λ*, Fig. 4 displays the desired traces. While the trace of 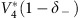 continues to lower bound the trace of the node voltage *V*_4_, we find in this case that Δ=0.75 when voltage collapse occurs for the coupled equations at *λ*/*λ*_collapse_=1. As expected, in this regime the unmodeled coupled power flow effects become crucial and the simplified decoupled model (1), on which our analysis is based, becomes invalid. Said differently, when reactive power demands in the network are low, our analytic prediction of the point of voltage collapse based on the simplified model (1) is overly optimistic. We comment further on extensions of our analysis to the coupled case in the ‘Discussion' section and in Supplementary Note 5.

**Figure 5:**
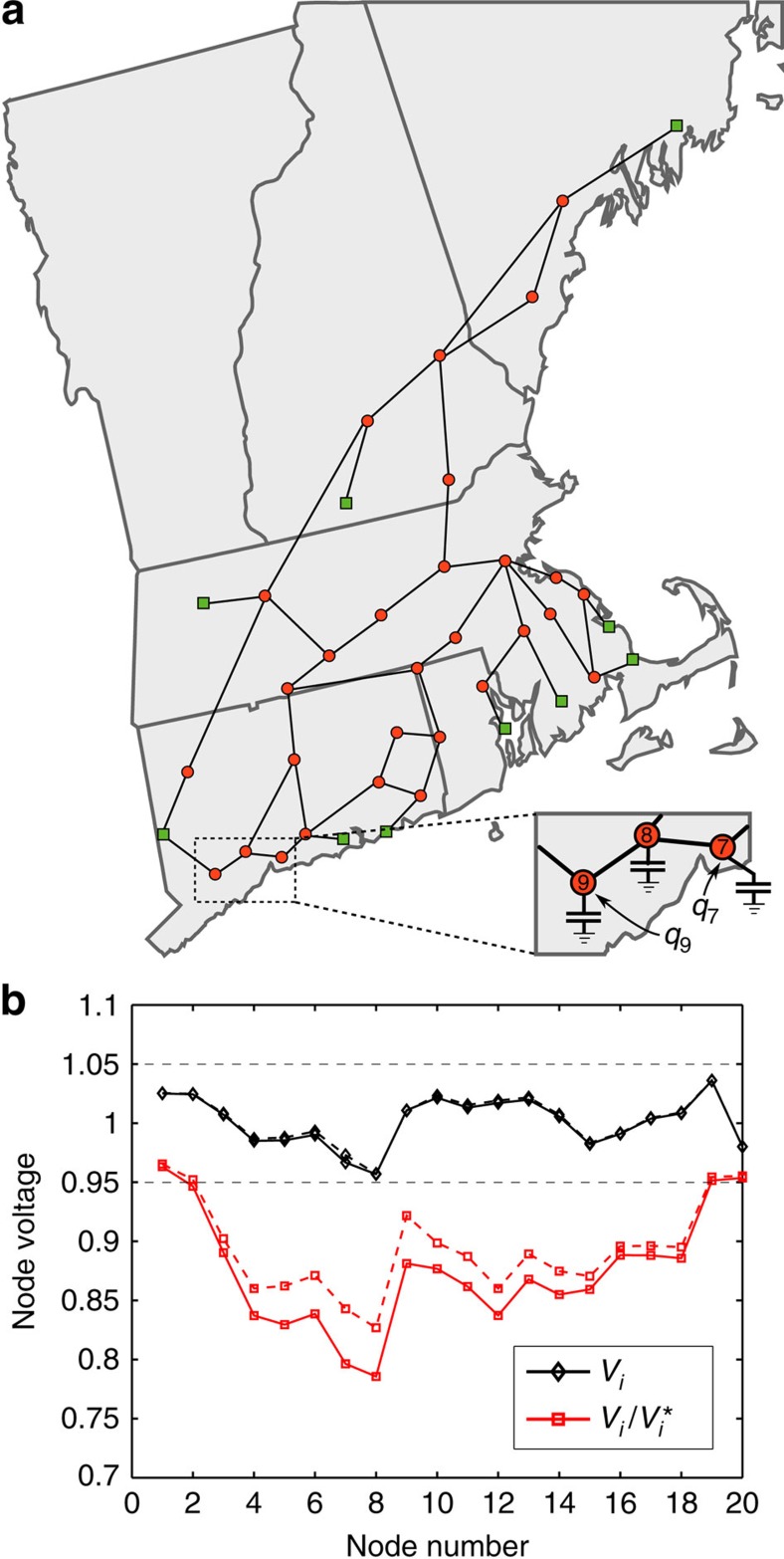
Corrective action results for the reduced New England 39-node network. (**a**) Depiction of the reduced New England grid. Load nodes {1,…,30} are red circles, while generators {31,…,39} are green squares. Shunt capacitors are present at all load nodes, but shown explicitly at nodes 7, 8 and 9. (**b**) Results of corrective action study. Voltage profile *V*_*i*_ (black) and scaled voltages 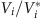 (red), before (solid) and after (dashed) corrective action. All voltages were scaled by the grid's base voltage *V*_base_=345 kV. Horizontal dashed lines are operational limits for *V*_*i*_ of ±5% from base voltage. For clarity only nodes {1,…,20} are plotted. Map by freevectormaps.com.

Our final study illustrates the use of our stability condition (7) for determining corrective actions, with the goal of increasing grid stability margins. The New England grid in Fig. 5a is experiencing peak loading conditions, and shunt capacitors have been switched in at all substations (red nodes) to support voltage magnitudes, keeping the voltage profile (solid black in Fig. 5b) within operational bounds (dotted grey). Node 8 is under particularly heavy loading with a poor power factor of 0.82, and additional shunt capacitors at nodes 7 through 9 have been used to support the voltages in this area. While all voltages are maintained within the operational bounds, we calculate using the condition (7) that Δ=0.64, indicating the network is actually under significant stress. This stress is also apparent by numerically solving the lossy coupled power flow equations plotting the ratio 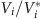 of the nodal voltage to the open-circuit voltage (solid red in Fig. 5b), as these ratios take into account the effects of shunt compensation; node 8 is experiencing the greatest stress. Consider the possibility of control equipment being present at the *i*th node of the network, capable of supplying an additional amount of reactive power *q*_*i*_ to the grid. Our goal is to select **q**=(*q*_1_,…,*q*_*n*_) to optimally increase grid stability margins. Such control could be realized actively through power electronic devices, or passively by curtailing local power consumption; in either case it is also desirable to minimize the total control action.

With this additional control capability, the stability metric (7) is modified to 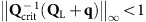. One immediately observes that the elements of 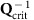 are providing information on where control action will be the most effective. For example, suppose that control equipment is present only at nodes seven and nine, but not at node eight (Fig. 5a). One finds for this example that 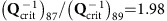, indicating that control action at node seven will be nearly twice as effective in reducing stress at node eight as the same control action would be if applied at node nine. From a purely topological viewpoint, this discrepancy in control sensitivity is surprising, as both nodes are neighbours of node eight. The stiffness matrix **Q**_crit_ incorporates not only the topology, but also the strength of connections between nodes, the locations of shunt capacitors and the relative proximity of generation (green nodes). Increasing *q*_7_ and *q*_9_ in this ratio provides the desired control action, allowing capacitor banks to be switched out, and we find that Δ=0.52 after control. A simple heuristic control has therefore reduced network stress by (0.64−0.52)/(0.52)≃23%, while the voltage profile of the grid (dotted black) is essentially unchanged.

In summary, the stability condition (7) can be simply and intuitively used to select control policies which increase grid stability margins with minimal control effort; additional details on eigenvector-based control directions^40^ and on the simulation setup are available in Supplementary Note 5 and the Supplementary Methods, respectively.

## Discussion

The stability condition (7) provides a long sought-after connection between network structure, reactive loading and the resulting voltage profile of the grid. As such, the condition (7) can be used to identify weak network areas and trace geographical origins of voltage instability by examining the entries of the vector 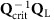. This allows for the effective placement of voltage control equipment, and the automatic dispatch of generation to mitigate voltage fluctuations, creating a self-healing network. The condition (7) can serve as a bridge between intuition-based heuristics for voltage control and more computational optimization approaches, and the use of (7) for systematic control design is currently under investigation.

The results reported here are a first step towards an analytic approach to assessing and strengthening the voltage stability of power grids. A limitation of the current work is that active power demands are included only implicitly in the condition (7), through the stiffness matrix **Q**_crit_ which contains the effective coupling weights *B*_*ij*_=*b*_*ij*_ cos(*θ*_*i*_−*θ*_*j*_). While our formal theoretical results hold only for the approximate model, the results of Table 1 show that this approximation is extremely accurate under normal operating conditions, and the results of Fig. 5 indicate that our framework provides effective control guidelines even when this assumption is violated. As can be seen from Fig. 4, however, this decoupling approximation tends to degrade near points of voltage collapse, where second-order effects due to active power flows become crucial, and the predictions of the simplified decoupled model and the coupled active/reactive power flow model diverge (Supplementary Note 5). The key direction for future work is therefore the development of a more advanced analytic test which explicitly includes active power demands and does not require that the stiffness matrix be updated as phase-angle differences *θ*_*i*_−*θ*_*j*_ change. This should allow for the rigorous extension of our theoretical results to coupled active/reactive power flow. Another limitation of model (1) is the assumption that resistances between nodes in the network are negligible. While this assumption is quite reasonable in large high-voltage transmission networks, resistances, nonetheless, generate additional voltage drops, and losses may become sizable due to large current flows as the network becomes stressed. Extending the stability test (7) to lossy power flow models is therefore another key step towards an analytic understanding of power flow. These two extensions are under investigation, and if completed will translate the new theoretical framework presented here into a robust set of analysis and design tools for practical power grids. We expect that a generalized stiffness matrix similar to equation (6) will play a key role in these more general problem setups.

An area where these results may have a major impact is in contingency screening, where system operators computationally assess failure scenarios to determine if the grid remains stable. Due to the low computational overhead of evaluating analytic conditions such as our stability condition (7), further developments of the theory may allow for the fast assessment of many more contingencies than is currently feasible, or a single condition could be derived which guarantees the stability of the system under all contingencies within a certain class. Finally, we note that similar matrix techniques for incorporating network structure should prove relevant in other complex networked systems displaying polynomial nonlinearities, such as ecological population models, chemical reaction networks, and viral epidemic spreading.

## Methods

### Main result derivation

The key step in deriving equation (7) is recognizing the physical significance of the open-circuit voltages 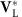 in equation (5). Physically, 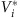 is the voltage one would measure at the *i*th node of the network when *Q*_1_=*Q*_2_=⋯=*Q*_*n*_=0. The condition (7) was derived by reformulating the power flow (1) as a fixed-point equation of the form **x**=**f**(**x**), where 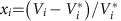 is a shifted and normalized voltage variable. With this notation, the power flow (1) takes the dimensionless form 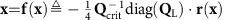, where 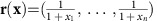. Imposing invariance of the set {*x* : |*x*_*i*_|≤*δ*, *i*=1,…,*n*} under the fixed-point map **f**(**x**) leads to condition (7). Existence and uniqueness of the equilibrium was shown by applying the contraction mapping theorem. Finally, stability was confirmed by showing that the Hessian matrix of the energy function is positive definite at the equilibrium (Supplementary Note 3 and 5).

### Properties of stiffness matrix

In all publicly available test cases, the sub-matrix **B**_LL_ is a nonsingular Metzler matrix. It follows that its inverse has nonpositive elements^41^, that the matrix 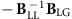 is nonnegative, and hence that the open-circuit voltages 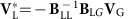 as defined in equation (5) are strictly positive. The stiffness matrix **Q**_crit_ used in the condition (7) inherits this Metzler property, and also posses an inverse with nonpositive elements. In particular, it holds that 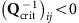 with strictly inequality if and only if there exists a path in the network between load node *i* and load node *j* which does not intersect any generator node. Thus, reactive loading at node *j* influences the voltage at node *i* and vice versa, even if nodes *i* and *j* are not one-hop neighbours. When there are multiple groups of loads electrically isolated from one another by generators, the stability test (7) therefore decouples into an identical test for each group.

### Numerical studies

Extensive details on the construction of our three numerical experiments may be found in the Supplementary Methods. All studies were implemented using the standard power flow solution techniques from the MATPOWER package^39^.

## Additional information

**How to cite this article:** Simpson-Porco, J. W. *et al*. Voltage collapse in complex power grids. *Nat. Commun.* 7:10790 doi: 10.1038/ncomms10790 (2016).

## Supplementary Material

Supplementary Information Supplementary Figures 1-4, Supplementary Notes 1-7, Supplementary Methods and Supplementary References.

## Figures and Tables

**Table 1:** Voltage stability condition applied to 11 test networks.

Numerical testing of theoretical predictions				
Test case (1,000 instances)	Condition correctness	Exact deviation (*δ*_exact_)	Predicted deviation (*δ*_−_)	Condition accuracy
9 bus system	True	5.50·10^−2^	5.52·10^−2^	3.56·10^−3^
14 bus system	True	2.50·10^−2^	2.51·10^−2^	1.96·10^−3^
RTS 24	True	3.28·10^−2^	3.29·10^−2^	3.28·10^−3^
30 bus system	True	4.72·10^−2^	4.75·10^−2^	7.64·10^−3^
New England 39	True	5.95·10^−2^	5.99·10^−2^	5.97·10^−3^
RTS ‘96 (2 area)	True	3.44·10^−2^	3.45·10^−2^	3.81·10^−3^
57 bus system	True	0.97·10^−1^	0.99·10^−1^	2.97·10^−2^
RTS ‘96 (3 area)	True	3.57·10^−2^	3.58·10^−2^	3.94·10^−3^
118 bus system	True	2.68·10^−2^	2.69·10^−2^	3.63·10^−3^
300 bus system	True	1.32·10^−1^	1.36·10^−1^	3.03·10^−2^
Polish 2,383 system	True	4.03·10^−2^	4.06·10^−2^	8.55·10^−3^

Condition correctness is whether the implication 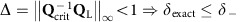 holds for every network realization, where 
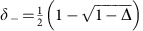
 and *δ*_exact_ is determined numerically. Exact and predicted deviations are averaged values of the respective quantities over all realizations. Condition accuracy is calculated as (*δ*_−_−*δ*_exact_)/*δ*_exact_, and averaged over 1,000 randomized instances for each network, with 30% of generation (resp. 30% of load) randomized by 30% (resp. 50%) using a normal distribution centred around base conditions.
