# Supplementary material for: Voltage collapse in complex power grids
Source: Nat Commun. 2016 Feb 18;7:10790. doi: 10.1038/ncomms10790 (PMC4759633; doi:10.1038/ncomms10790)
Supplement: Supplementary Information — Supplementary Figures 1-4, Supplementary Notes 1-7, Supplementary Methods and Supplementary References. [file ncomms10790-s1.pdf]

Supplementary Information

Supplementary Figures

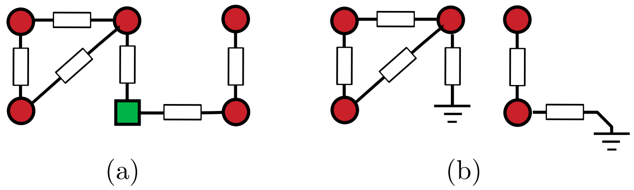

Supplementary Fig. 1: Example of original and induced networks. (a) An example network with one generator (green square) and five loads (red circles); (b) The subgraph induced by the load nodes $\mathcal{V}_L$. Generator buses separate the induced load subgraph into disconnected components in which voltage stability can be assessed independently.

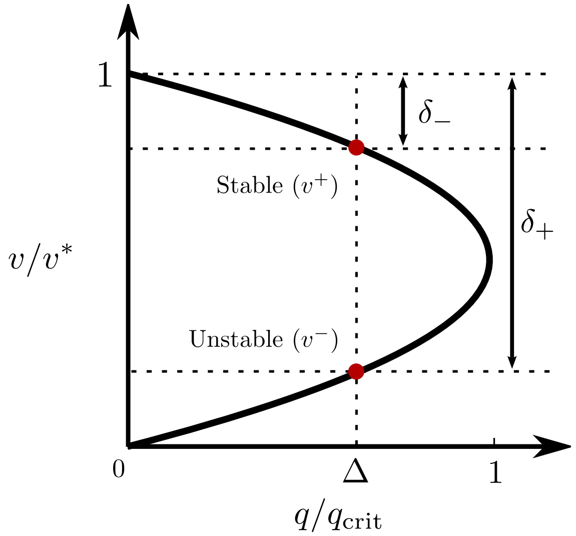

Supplementary Fig. 2: Locus of solutions to the one-dimensional power flow equation (20). The stable solution v^+ lies on the top portion of the curve, deviating from the open-circuit voltage v^* by a small percentage δ_-. The unstable solution lies on the bottom portion of the curve, deviating from v^* by a large percentage δ_+.

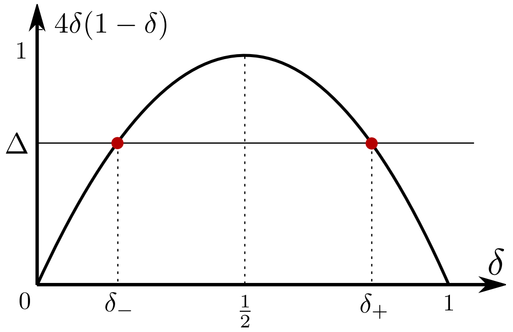

Supplementary Fig. 3: Illustration of the construction leading to the parametric condition (31).

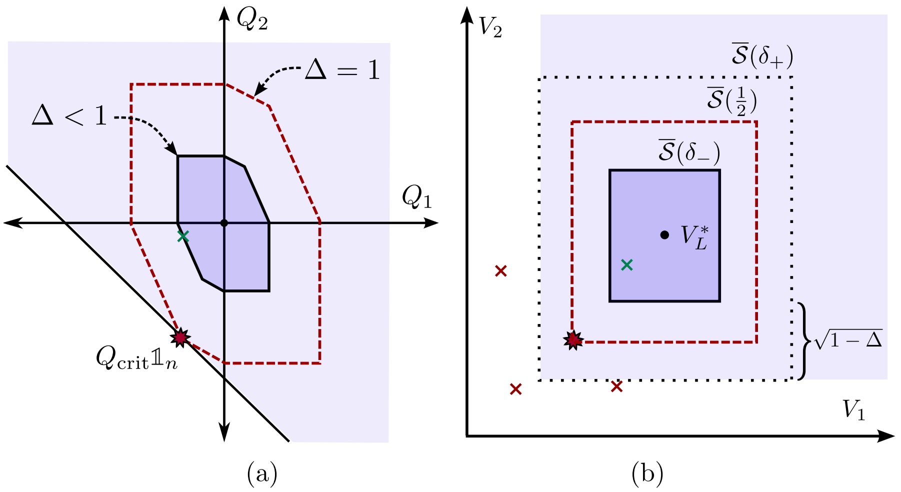

Supplementary Fig. 4: Visualization of Supplementary Theorem 1 when $\Delta \in]0,1[$. (a) The partitioning of load-space. The critical load profile which lies on the existence boundary is marked with a red star in (a), and leads to the voltage solution marked with a star in (b). The light shaded region above the diagonal line corresponds to the necessary solvability condition in Proposition 2. The dark shaded region is the convex hull of load profiles which satisfy $\Delta = 4\delta_\pm(1-\delta_\pm)$. (b) The partitioning of voltage-space. Red crosses (×) denote unstable power flow equilibria, while the green cross (×) indicates the stable equilibrium. The dark shaded region is the stability set $\overline{\mathcal{S}}(\delta_-)$ in which the unique voltage-stable solution is guaranteed to exist, while the light shaded region is the open set of Supplementary Theorem 1 2), where solutions are forbidden. The Venikov index $K_{\text{V}} = \sqrt{1-\Delta}$ provides a guaranteed bound between stable and unstable equilibria in voltage-space.

Supplementary Notes

Supplementary Note 1 – Organization and Preliminaries

This supplementary information is organized as follows.

Supplementary Note 1 — Organization and Preliminaries — provides a table of contents for this supplementary information and establishes some mathematical notation.

Supplementary Note 2 — *Review of Explicit Voltage Stability Conditions* — reviews the parametric voltage stability conditions available in the literature.

Supplementary Note 3 — *Mathematical Models* — provides a detailed description of the considered power network models, including transmission grid, generator, and load modeling. We also describe the approximations which lead to the reactive power flow model considered in the main article.

Supplementary Note 4 — *Energy, Voltage Stability & The Power Flow Jacobian* — formally defines the concept of long-term voltage stability, and relates voltage stability of an operating point to the local minima of the energy function and to the non-singularity of the power flow Jacobian.

Supplementary Note 5 — *Voltage Stability in Complex Power Networks* — contains the mathematical analysis leading to the main result presented in the article. We offer interpretations and comments throughout along with several corollaries.

Supplementary Note 6 — *Monotonicity of Stability Margins With Respect to Parameters: Results and Counterexamples* — examines how variations in grid parameters influence the proposed stability margin of the grid.

Supplementary Note 7 — *Voltage Stability Condition Incorporating Generator Injection Limits* — presents a generalized version of our main result which accounts for generator reactive power limits.

Finally, Supplementary Methods provides additional details regarding the extensive numerical simulations presented in the main article.

Sets, vectors and matrices: Given a finite set $\mathcal{V}$, let $|\mathcal{V}|$ denote its cardinality. The set $\mathbb{R}$ (resp. $\mathbb{R}_{\geq 0}$, $\mathbb{R}_{>0}$) is the field of real (resp. nonnegative real, strictly positive real) numbers, and $\mathbb{C}$ is the field of complex numbers. For $x \in \mathbb{R}^n$, $[x] \in \mathbb{R}^{n\times n}$ is the associated diagonal matrix. Given $x, y \in \mathbb{R}^n$, we write $x \geq y$ if $x_i \geq y_i$ for each $i \in \{1, \ldots, n\}$, and will occasionally write $z \in [x, y]$ if $x \leq z \leq y$. Similarly we define $x > y$ and $z \in \,]x, y[$ in the obvious ways. We let $\mathbb{1}_n$ and $\mathbb{0}_n$ be the n-dimensional column vectors of unit and zero entries, with I_n the $n \times n$ identity matrix. For nonempty sets $\mathcal{K}_1$ and $\mathcal{K}_2$, $\mathcal{K}_1 \setminus \mathcal{K}_2 = \{x \in \mathcal{K}_1 \mid x \notin \mathcal{K}_2\}$ is the set of elements in $\mathcal{K}_1$ which do not also belong to $\mathcal{K}_2$.

M-Matrices: A matrix $A \in \mathbb{R}^{n\times n}$ is a Z-matrix if $A_{ij} \leq 0$ for all $i \neq j$. The *spectral radius* $\rho(A)$ of a real-valued matrix $A \in \mathbb{R}^{n\times n}$ is $\rho(A) = \max\{|\lambda|_{\mathbb{C}} \mid \det(\lambda I_n - A) = 0\}$, where $|x|_{\mathbb{C}}$ is the magnitude of $x \in \mathbb{C}$. A Z-matrix $A \in \mathbb{R}^{n\times n}$ is an M-matrix if it can be expressed as $A = sI_n - B$, where $B \in \mathbb{R}^{n\times n}$ has nonnegative elements and $s \geq \rho(B)$. If A is a nonsingular M-matrix, then the elements of A^{-1} are nonnegative [1]. Moreover, if the directed graph induced by the sparsity pattern of A is strongly connected, then A is irreducible and the elements of A^{-1} are all strictly positive [2, 1].

Geometry on the circle: The set $\mathbb{S}^1$ is the *unit circle*, an *angle* is a point $\theta \in \mathbb{S}^1$, and an *arc* is a connected subset of $\mathcal{S}^1$. The *geodesic distance* between two angles $\theta_1, \theta_2 \in \mathcal{S}^1$, denoted by $|\theta_1 - \theta_2|$,

is the minimum length of the counter-clockwise and clockwise arcs connecting θ_1 and θ_2.

The ∞-Norm and Unit Balls: The ∞-norm of $x \in \mathbb{R}^n$ is given by $\|x\|_\infty = \max_{i\in\{1,\ldots,n\}} |x_i|$, and the induced norm of a matrix $A \in \mathbb{R}^{n\times n}$ is $\|A\|_\infty = \max_{i\in\{1,\ldots,n\}} \sum_{j=1}^{n} |A_{ij}|$. It holds that $\rho(A) \leq \|A\|_\infty$. The associated closed unit ball is defined by $\overline{\mathcal{B}}_\infty(\delta) \triangleq \{x \in \mathbb{R}^n \mid \|x\|_\infty \leq \delta\}$, with $\mathcal{B}_\infty(\delta)$ being its interior.

Nonlinear Equations: For a smooth map $f : \mathbb{R}^n \to \mathbb{R}^n$, a solution $x^* \in \mathbb{R}^n$ of $f(x) = \mathbb{0}_n$ is a *regular* solution if the Jacobian matrix $\frac{\partial f}{\partial x}(x^*)$ is nonsingular. For a compact set $X \subset \mathbb{R}^n$, a map $f : X \to X$ is a *contraction map* on X if there exists an $\alpha \in [0, 1[$ (the *contraction rate*) and a norm $\|\cdot\|$ such that for any $x, y \in X$, $\|f(x) - f(y)\| \leq \alpha\|x - y\|$. If in addition X is convex and f is continuously differentiable, then f is a contraction map on X with contraction rate α if $\|\frac{\partial f}{\partial x}(x)\|_{\mathrm{ind}} < \alpha$ for each $x \in X$, where $\|\cdot\|_{\mathrm{ind}}$ is the matrix norm induced by the vector norm $\|\cdot\|$.

Supplementary Note 2 – Review of Explicit Voltage Stability Conditions

In [3, 4, 5] the completely decoupled ($\gamma = 0$ in Assumption 3) power flow equations were analyzed using fixed point techniques, resulting in vector-valued sufficient conditions of the form $h_i(V^{\min}) \leq Q_i \leq h_i(V^{\max})$ for all $i \in \mathcal{L}$, where $V^{\min}$ and $V^{\max}$ are upper and lower bounds on the nodal load voltages. No guarantees are given on uniqueness of the stable equilibrium, the analysis is not clearly related to the network structure, and the Jacobian must satisfy a diagonal dominance condition, which is known to be restrictive and often violated when including line charging capacitors and capacitive shunts [6]. Moreover, [3] requires an unrealistic tiering assumption on the network structure, where all load (PQ) buses are at most once-removed from a generator (PV) bus. In [6] the network stiffness was quantified by the product of the smallest eigenvalue (in magnitude) of the admittance matrix $|\lambda_1(B_{LL})|$ and a desired lower bound on the voltage at any load bus, with the severity of loading captured by the largest load $\|Q_L\|_\infty = \max_{i\in\mathcal{L}} |Q_i|$. This conservative sufficient condition heavily underestimates the network stiffness while overestimating the severity of loading, and does not take into account the interaction between network structure and the spatial distribution of load. The recent work [7] shows that a similar condition suffices for solvability of the coupled active/reactive power flow equations, but in distribution networks with a single generator. Other results on power flow solvability and security [8, 9, 10, 11] provide only necessary solvability conditions, or use constant-impedance/current load models, and are therefore unable to assess voltage collapse.

The papers [12, 13] provide branch-wise necessary conditions for voltage collapse, showing that voltage collapse can occur only after at least one branch of the network is saturated past a limit, termed the *static transfer stability limit.* While this is an insightful analysis framework, evaluating the transfer limit conditions requires knowledge of branch-wise power flows and voltage magnitudes, that is, the conditions are checked based on the output of a power flow program. In contrast, our goal is to work directly from the given data of the problem (topology, impedances, loading, and generator voltages) and develop conditions which guarantee the existence of a power flow solution and characterize its robustness margins.

Supplementary Note 3 – Mathematical Models

In this section we introduce the relevant power network models for the transmission network, generators, and loads. Our focus is on high-voltage power transmission networks.

Transmission Network Modeling

Throughout this work we consider a connected, phase-balanced power network operating in sinusoidal quasisteady-state. The network is modeled as a weighted and undirected graph $G(\mathcal{V}, \mathcal{E}, Y)$ with nodes (or *buses*) $\mathcal{V}$, edges (or *branches*) $\mathcal{E} \subset \mathcal{V} \times \mathcal{V}$, and complex edge weights (or *admittances*) $y_{ij} \in \mathbb{C}$. The network has two distinct types of buses: loads $\mathcal{L}$ and generators $\mathcal{G}$, such that $\mathcal{V} = \mathcal{L} \cup \mathcal{G}$. For notational simplicity, we set $n \triangleq |\mathcal{L}|$, $m \triangleq |\mathcal{G}|$ and assume $n, m \geq 1$. To each bus we associate a phasor voltage $U_i = V_i e^{\mathrm{j}\theta_i} \in \mathbb{C}$ where $V_i \geq 0$ is the voltage magnitude and $\theta_i \in \mathbb{S}^1$ is the voltage angle, and a complex power injection $S_i = P_i + \mathrm{j}Q_i$.

Transmission lines are represented using the standard lumped parameter *Π-model*, which allows for the inclusion of inductive/capactive shunts, tap-changing transformers, and line charging capacitors [14]. We encode the weights and topology in the bus admittance matrix $Y \in \mathbb{C}^{(n+m)\times(n+m)}$, with elements $Y_{ij} = -y_{ij}$ and $Y_{ii} = -\sum_{j=1}^{n+m} y_{ij} + y_{\mathrm{shunt},i}$, where $y_{\mathrm{shunt},i}$ is the shunt element at bus i. The conductance matrix G and susceptance matrix B are defined by $G = \mathrm{Re}(Y)$ and $B = \mathrm{Im}(Y)$. For the 100+ kV transmission-level networks we consider, the admittances of the transmission lines are dominantly inductive. The real part of the bus admittance matrix is therefore negligible, and $Y \simeq \mathrm{j}B$ [14]; see [15, 16] for studies concerning lossy models.

For later use we summarize for properties of the susceptance matrix.

Fact 1 (Properties of Susceptance Matrix [5]). *If the network contains no phase-shifting transformers and the transmission lines are not overcompensated by series capacitors, then for all $i, j \in \mathcal{V}$*

(i) **Symmetry:** $B_{ij} = B_{ji}$;

(ii) **Sign Structure:** $B_{ij} \geq 0$, *with* $B_{ij} > 0$ *if and only if* $\{i, j\} \in \mathcal{E}$;

(iii) **Self-susceptances:** $B_{ii} = -\sum_{j=1, j\neq i}^{n+m} B_{ij} + B_{i,\mathrm{shunt}}$ *for all* $i \in \mathcal{V}$, *where* $B_{i,\mathrm{shunt}}$ *is the shunt element at node* i. *The shunt at node* i *is capacitive if* $B_{i,\mathrm{shunt}} > 0$, *and inductive if* $B_{i,\mathrm{shunt}} < 0$.

Partitioning the susceptance matrix according to load and generators as

$$B = \begin{pmatrix} B_{LL} & B_{LG} \\ B_{GL} & B_{GG} \end{pmatrix}, \tag{1}$$

our results to follow require the following weak assumptions on the sub-matrix B_{LL} (sometimes referred to as a *grounded* susceptance matrix) and the network topology.

Assumption 1 (Susceptance Matrix). *The (negative) grounded susceptance matrix $-B_{LL} \in \mathbb{R}^{n\times n}$ is a nonsingular M-matrix.*

Assumption 2 (Connected Subgraph). *The subgraph of $G(\mathcal{V}, \mathcal{E}, B)$ induced by the load nodes $\mathcal{L}$ is connected.*

Assumption 1 is universally satisfied in practical networks [6, Section III], and always satisfied in the absence of line charging and shunt capacitors due to diagonal dominance [17]. Assumption 2 may be taken without loss of generality when studying the fundamental physics of reactive power flow in transmission networks, as the voltage-regulated generator buses electrically isolate groups of load buses from one another (Supplementary Fig. 1). Note that under Assumptions 1 and 2, $-B_{LL}$ is a symmetric, irreducible nonsingular M-matrix, and hence all elements of $-B_{LL}^{-1}$ are strictly positive [2, 1].

Applying Kirchoff's and Ohm's Laws to the network, the vector of nodal complex power injections $S = (S_1, \ldots, S_{n+m})$ is related to the susceptance matrix B and the vector of nodal complex voltages $U = (U_1, \ldots, U_{n+m})$ by $S = [U] \cdot \mathrm{conj}(YU) = [U] \cdot \mathrm{conj}(jBU)$, where $\mathrm{conj}(x)$ is the element-wise complex conjugation of the vector $x \in \mathbb{C}^{n+m}$. In components, the real and imaginary parts of the product $[U] \cdot \mathrm{conj}(jBU)$ define the *power flow functions* $g_i, h_i : \mathbb{R}^{n+m} \times \mathbb{T}^{n+m} \to \mathbb{R}$, which can be quickly calculated to be

$$g_i(V, \theta) \triangleq \sum\nolimits_{j=1}^{n+m} V_i V_j B_{ij} \sin(\theta_i - \theta_j) \,, \tag{2a}$$

$$h_i(V, \theta) \triangleq -\sum\nolimits_{j=1}^{n+m} V_i V_j B_{ij} \cos(\theta_i - \theta_j) \,. \tag{2b}$$

Physically, g_i and h_i are the *active power* and *reactive power* injected at node $i \in \mathcal{V}$ when the voltage magnitudes and angles are $V = (V_1, \ldots, V_{n+m})$ and $\theta = (\theta_1, \ldots, \theta_{n+m})$.

Generator Modeling

As standard in power flow analysis, the synchronous generators at nodes $\mathcal{G}$ are modeled as *PV buses*, at which the active power injections $P_i \in \mathbb{R}$ are fixed by the prime movers and voltage magnitudes $V_i > 0$ are tightly regulated by Automatic Voltage Regulators (AVR). This regulation is always achieved under normal operating conditions, when field and stator currents have not reached their operational limits [18, Chapter 3.3.5]. In the saturated case, the generator reaches its operational reactive power limit. One approach to handling this saturation is to change the generator model – reactive power output is held fixed at the operational maximum, and the terminal voltage is allowed to float. In this modeling framework, the generator behaves as a negative constant-power load [19, 20]. A downside of this approach is that the specific limits encountered depend on the direction of the loading vector in the space of parameters. In Supplementary Note 6 we take a different approach and instead present a condition under which generators meet their reactive power overexcitation limits, thereby avoiding this issue. Our results can also be extended to include distributed generation interfaced through power inverters with voltage-droop controllers [21, 22] or microgenerators which inject constant amounts of current or power (modeled as negative loads). Moreover, our results are not dependent on the inclusion of generator dynamics due to time-scale separation: the transient instabilities associated with generator swing and flux decay dynamics occur on the order of seconds [14], while the voltage collapse phenomena we consider occur over minutes [23].

Load Modeling

Load models characterize the power consumed by individual or aggregate loads, while taking into account relevant voltage and frequency-dependent behavior and/or dynamic phenomena. For studying voltage collapse, we are concerned only with the long-term feasibility of the network operating point, and therefore with no loss of generality may restrict ourselves to static load models [24, 25]. As such, the load models we consider have no internal state variable, and the active and reactive power demands $P_i, Q_i \in \mathbb{R}$ at load node $i \in \mathcal{L}$ are expressed as static functions $P_i(V_i)$ and $Q_i(V_i)$ of the local bus voltage magnitude. Kirchhoff's Current Law requires that power injections given by (2a)–(2b) equal the power demands $P_i(V_i)$ and $Q_i(V_i)$ at each node, yielding the power flow equations

$$P_i(V_i) = g_i(V, \theta)\,, \quad i \in \mathcal{L} \cup \mathcal{G}\,, \tag{3a}$$

$$Q_i(V_i) = h_i(V, \theta)\,, \quad i \in \mathcal{L}\,. \tag{3b}$$

We focus on the n *reactive power flow equations* (RPFE) (3b); see [26, 27] and the references therein for detailed analyses of the active power flow equations (3a). While in the main article we considered constant power loads $Q_i(V_i) = Q_i$, here we consider the more general "ZIP" load model [14]

$$Q_i(V_i) = b_{\mathrm{shunt},i} V_i^2 + I_{\mathrm{shunt},i} V_i + Q_i\,, \quad i \in \mathcal{L}\,, \tag{4}$$

which approximates the steady-state behavior of a wide class of practical loads. The model (4) is a concatenation of three separate loads:

(a) "Z"-load: a shunt connection to ground through a susceptance $b_{\mathrm{shunt},i} \in \mathbb{R}$.

(b) "I"-load: a constant current device injecting a reactive current $I_{\mathrm{shunt},i} \in \mathbb{R}$. The current is *leading* if $I_{\mathrm{shunt},i} > 0$ and *lagging* if $I_{\mathrm{shunt},i} < 0$;

(c) "P"-load: a constant power device, injecting fixed reactive power $Q_i \in \mathbb{R}$. The load is called *capacitive* if $Q_i > 0$, and *inductive* if $Q_i < 0$.

As we have already allowed for shunt loads in the admittance matrix B, we will without loss of generality set $b_{\mathrm{shunt},i} = 0$ in (4) for all $i \in \mathcal{L}$. In practice, the literature has established that the constant-power load model (c) is the most relevant one for steady-state security analysis [28], and the one most relevant from the perspective of both classic [18] and modern [11, 29] power system operation. This "stiff" (i.e., voltage independent) behavior may arise due to on-load tap-changing transformers maintaining a constant voltage at the load supply point, or as an estimate of aggregate load as in utility forecasts [28]. It can be shown that when the constant-power portion of the load model (4) is zero, the reactive power balance (3b) degenerates into a system of linear equations in the voltage magnitudes [30, 9, 10]. Moreover, it has been noted that constant power load modeling is generic for feasibility studies in the sense that it tightly captures the transfer limitations of the network, and allows the study of whether an operating point exists *for any* static or dynamic load model, when the load consumes a specified amount of power [31]. If a feasible operating point can be determined, it can then be used as part of the initial conditions for further numerical dynamic stability studies. See [32, 33, 9, 21] for additional information and analysis.

Power Angle Decoupling

In practice, typical solutions to the power flow equations (3a)–(3b) have the property that $|\theta_i - \theta_j| \leq \gamma$ for each edge $\{i,j\} \in \mathcal{E}$ and some small value of $\gamma > 0$, and that load voltage magnitudes V_i are roughly equal to the generator voltage level. This leads to an important (in)sensitivity relationship for reactive power flow, which we now describe. Evaluating the derivative of the reactive power injection (2b) around such a solution, we find that

$$\left|\frac{\partial h_i}{\partial \theta_k}\right| = V_i V_k B_{ik} |\sin(\theta_i - \theta_k)| \leq V_i V_j B_{ij} \sin(\gamma) \simeq 0\,, \qquad k \neq i\,, \tag{5a}$$

$$\left|\frac{\partial h_i}{\partial V_k}\right| = V_i B_{ij} |\cos(\theta_i - \theta_k)| \geq V_i B_{ik} \cos(\gamma) \simeq V_i B_{ik}\,, \qquad k \neq i\,. \tag{5b}$$

with similar formulas holding for the diagonal elements. It follows that the reactive power injections (2b) are insensitive to changes in the power angles $\theta_i - \theta_j$ around such solutions, and that active power enters through second order effects. It is therefore common to study (3b) under a *decoupling* assumption, in which the power angles are treated as parameters [6, 30], or even assumed to be negligible [4, 5]. We formalize these qualitative statements into the following technical assumption.

Assumption 3 (Power Angle Decoupling)**.** *The power angles are constant and such that* $|\theta_i - \theta_j| \leq \gamma$ *for some value* $\gamma \in [0, \pi/2[$ *and for all branches* $\{i,j\} \in \mathcal{E}$ *of the network.* □

See [5] and Ref. 14 of [30] for analysis on the error introduced by total decoupling, that is, the extreme case $\gamma = 0$ in Assumption 3. In practical networks, a typical value of γ in Assumption 3 would be 5°. Under Assumption 3, from the form of (2b) it is clear that we can define an *effective susceptance matrix* by grouping the original line susceptances B_{ij} and the power angle terms $\cos(\theta_i - \theta_j)$. The properties of the original susceptance matrix from Fact 1 also hold for the effective susceptance matrix, and one may verify that if Assumption 1 holds, then the corresponding submatrix of the effective susceptance matrix is also a nonsingular M-matrix. To keep notation simple, in what follows we will denote by B the effective susceptance matrix with elements $B_{ij}\cos(\theta_i - \theta_j)$ for $i, j \in \mathcal{V}$. With this notation, the power flow equation (3b) becomes

$$Q_i + V_i I_{\mathrm{shunt},i} = -\sum\nolimits_{j=1}^{n+m} V_i B_{ij} V_j\,, \qquad i \in \mathcal{L}\,. \tag{6}$$

The Reactive Power Flow Equations

In vector notation, the reactive power flow equation (6) can be written as

$$Q_L + [V_L] I_{\mathrm{shunt}} = -[V_L]\left(B_{LL} V_L + B_{LG} V_G\right)\,, \tag{7}$$

where $Q_L = (Q_1, \ldots, Q_n)$ is the vector of constant power load demands, $I_{\mathrm{shunt}} = (I_{\mathrm{shunt},1}, \ldots, I_{\mathrm{shunt},n})$ is the vector of constant current load demands, $V_L = (V_1, \ldots, V_n) > \mathbb{0}_n$ is the vector of load voltage magnitudes, $V_G = (V_{n+1}, \ldots, V_{n+m}) > \mathbb{0}_m$ is the vector of constant generator voltage magnitudes, $[V_L]$ is the diagonal matrix of load voltages. Equivalently, we may write

$$\boxed{Q_L = -[V_L] B_{LL} (V_L - V_L^*)\,,} \tag{8}$$

where we have defined the *open-circuit load voltages* $V_L^* \in \mathbb{R}^n$ by

$$V_L^* \triangleq -B_{LL}^{-1}(B_{LG}V_G + I_{\text{shunt}})\,. \tag{9}$$

The terminology open-circuit means that V_i^* is the voltage measured at load bus $i \in \mathcal{L}$ when the constant-power loads are open-circuited, that is, $Q_1 = Q_2 = \cdots = Q_n = 0$. We make the following standing assumption regarding the constant current portion I_{shunt} of the load.

Assumption 4 (Shunt Current Restriction)**.** *The shunt currents are not overly inductive. In particular,* $B_{LG}V_G + I_{\text{shunt}} > \mathbb{0}_n$*.*

Assumption 4 always holds if the loads draw capacitive currents ($I_{\text{shunt}} \geq \mathbb{0}_n$), and is always met in practical networks. This assumption leads to positive open-circuit voltages.

Lemma 1 (Open-Circuit Voltages)**.** *Under Assumption 4, the open-circuit load voltages are strictly positive. That is,* $V_L^* > \mathbb{0}_n$*.*

Proof: By Assumptions 1 and 2, the open-circuit voltages (9) are well defined and each element of $-B_{LL}^{-1}$ is positive. Since $B_{LG}V_G + I_{\text{shunt}} > \mathbb{0}_n$, every element of (9) is therefore strictly positive. □

Equation (8) is our preferred formulation of the RPFE (3b)–(4) since it highlights the tendency of the load voltages V_L to align with their open-circuit values V_L^*.

Supplementary Note 4 – Energy, Voltage Stability & The Power Flow Jacobian

The term *voltage stability* has been appropriated over decades of research to refer to many different static and/or dynamic stability concepts, ranging orders of magnitude in time-scales. Herein we make use of the long-term quasi static notion of voltage stability [34, 35], which corresponds to the way the high-voltage solution of a power network should change under small load perturbations during regular operation. This is the notion of stability most relevant for guarding against static voltage instability collapse.

Definition 1 (Local Voltage Stability)**.** *A regular solution* $V_L \in \mathbb{R}^n$ *of the RPFE* (8) *is*

(i) locally voltage-stable if for each pair of load buses $i, j \in \mathcal{L}$*,*

$$\frac{\partial V_i}{\partial Q_j} > 0\,, \tag{10}$$

(ii) voltage-unstable if it is not locally voltage-stable.

Since a decreasing value of Q_j in (10) corresponds to an *increasing inductive load*, voltage stability is an "increasing load, decreasing voltage" condition. Definition 1 is the stability definition implicitly used when discussing stability margins derived from Jacobian-based voltage stability indices, and is the relevant stability concept at long time-scales on the order of minutes [23, 36]. Long-term voltage

stability is time-scale separated from instabilities induced by fast generator or load dynamics, and can therefore be studied separately [28].

The energy function introduced in the main article is particularly useful for understanding voltage stability as in Definition 1. The following result relates the energy function, voltage stability, and the Jacobian matrix of the power flow equation.

Lemma 2 (Energy, Stability, & The Power Flow Jacobian)**.** *Consider the reactive power flow equation* (6)*, its Jacobian matrix* $J(V_L) \in \mathbb{R}^{n\times n}$ *being given by*

$$J(V_L) = [V_L]B_{LL} + [B_{LL}(V_L - V_L^*)]\,, \tag{11}$$

and the energy function $E : \mathbb{R}^n_{>0} \to \mathbb{R}$ *defined by*

$$E(V_L) = \frac{1}{2}\sum_{i=1}^{n}\sum_{j=i+1}^{n+m} B_{ij}(V_i - V_j)^2 - \sum_{i=1}^{n}\left(\frac{1}{2}\kappa_i V_i^2 + I_{\mathrm{shunt},i}V_i + Q_i\ln(V_i)\right)\,, \tag{12}$$

where $\kappa_i \triangleq \sum_{j=1}^{n+m} B_{ij}$*. Suppose* $V_L > \mathbb{0}_n$ *is a regular solution of the RPFE* (6)*. Then the following statements are equivalent:*

(i) V_L *is a local minimum of the energy function* (12)*;*

(ii) V_L *is locally voltage-stable in the sense of Definition 1;*

(iii) the Jacobian $J(V_L)$ *given by* (11) *is Hurwitz.*

Proof: ***(i)*** $\Longleftrightarrow$ ***(iii)*** **:** The critical points of $E(V_L)$ satisfy $\partial E/\partial V_L = \mathbb{0}_n^T$, or in components for $k \in \mathcal{L}$

$$\frac{\partial E}{\partial V_k} = 0 = \sum_{j=1}^{n+m} B_{kj}(V_k - V_j) - \kappa_k V_k - I_{\mathrm{shunt},k} - \frac{Q_k}{V_k}\,. \tag{13}$$

After substituting for κ_k and simplifying, this becomes

$$0 = -Q_k - I_{\mathrm{shunt},k}V_k - V_k\sum\nolimits_{j=1}^{n+m} B_{kj}V_j\,, \tag{14}$$

which is the RPFE (6). It follows that the critical points of (12) are in one-to-one correspondence with the solutions of (6). Some simple calculations show that the components of the Hessian matrix $H(V_L)$ of $E(V_L)$ are given by

$$H_{k\ell}(V_L) = \frac{\partial^2 E}{\partial V_k \partial V_\ell} = -B_{k\ell} - \delta_{k\ell}\frac{Q_k}{V_k^2}\,, \qquad k,\ell \in \mathcal{L}\,, \tag{15}$$

where $\delta_{k\ell} = 1$ if $k = \ell$, and is zero otherwise. Substituting for Q_k from the power flow equation (6), we arrive at

$$H_{k\ell}(V_L) = -B_{k\ell} - \delta_{k\ell}\frac{1}{V_k}\left(I_{\mathrm{shunt},k} + \sum\nolimits_{j=1}^{n+m} B_{kj}V_j\right)\,, \tag{16}$$

which in matrix notation reads as

$$H(V_L) = -B_{LL} - [V_L]^{-1}[B_{LL}V_L + B_{LG}V_G + I_{\rm shunt}] \tag{17a}$$

$$= -B_{LL} - [V_L]^{-1}[B_{LL}(V_L - V_L^*)]\,, \tag{17b}$$

where we have used (9). By comparison, we see that the Hessian $H(V_L)$ is exactly $-[V_L]^{-1}$ times the Jacobian matrix (11) of the RPFE (8). It follows that the Hessian matrix is positive definite if and only if the Jacobian (11) is Hurwitz.

***(iii)* $\Longrightarrow$*(ii)* :** By definition the Jacobian matrix $J(V_L)$ relates infinitesimal changes in nodal voltage to corresponding changes power injections via

$$\frac{\partial Q_L}{\partial V_L} = -J(V_L)\,. \tag{18}$$

The matrix representation (11) can be obtained from (8), either by a calculation in components or via standard matrix identities. Note from (11) that for $i, j \in \mathcal{L}$, $J_{ij}(V_L) = V_i B_{ij} \geq 0$. Thus, $-J(V_L)$ is a Z-matrix. Defining the symmetric matrix $M \triangleq B_{LL} + [V_L]^{-1}[B_{LL}(V_L - V_L^*)]$, note that we may write $J(V_L) = [V_L]M$. Since $V_L > \mathbb{0}_n$, the generalized Courant-Fischer Theorem [37] then implies that all eigenvalues of $J(V_L)$ are real. So $-J(V_L)$ is a Z-matrix with real eigenvalues, and it follows then from [2, Item C_9] that $J(V_L)$ is Hurwitz if and only if $-J(V_L)$ is a nonsingular M-matrix. Moreover, these equivalent conditions hold true if and only if the inverse matrix $-J(V_L)^{-1}$ exists and has nonnegative elements [2, Item F_{15}]. By Assumption 2 it holds that $J(V_L)$ is irreducible, so $J(V_L)$ is in fact Hurwitz if and only if $-J(V_L)^{-1}$ exists and has strictly positive elements [1]. Since V_L is a regular solution, the Inverse Function Theorem [38, Chapter 9] states that there exists an open neighborhood $\mathcal{U}$ around V_L (resp. an open neighborhood $\mathcal{W}$ around Q_L) and a smooth function $G : \mathcal{W} \to \mathcal{U}$ such that $V_L = G(Q_L)$ for all $Q_L \in \mathcal{W}$. Moreover, the Jacobian of G satisfies

$$\frac{\partial G}{\partial Q_L} = \frac{\partial V_L}{\partial Q_L} = -J(V_L)^{-1}\,, \tag{19}$$

where in the last equality we used the fact that Q_L appears linearly in (8). Since $J(V_L)$ is Hurwitz, each element of $-J(V_L)^{-1}$ is strictly positive, and we conclude that V_L is locally voltage-stable.

***(ii)* $\Longrightarrow$*(iii)* :** Proceed by contraposition and suppose that $J(V_L)$ is not Hurwitz. Then by the set of implications preceding sufficiency, either a) there exists $i, j \in \mathcal{L}$ such that the ijth element of $-J(V_L)^{-1}$ is nonpositive, or b) $J(V_L)^{-1}$ does not exist. In the first case, this means precisely that $\partial V_i/\partial Q_j \leq 0$, so V_L is by definition voltage-unstable. The second case in which $J(V_L)$ is singular cannot occur, because V_L is a regular solution. □

Remark 1 (The Energy Function & Dynamic Stability)**.** *Voltage stability as considered in Definition 1 is consistent with local exponential stability of any dynamic load model which attempts to regulate power consumption to a constant value by demanding additional current under a drop in terminal voltage [28]. A simple example of this is the dynamic shunt susceptance model [33]* $\dot{b}_{\mathrm{shunt},i} = Q_i - b_{\mathrm{shunt},i}V_i^2$*, where the shunt susceptance* $b_{\mathrm{shunt},i}$ *at node* $i \in \mathcal{L}$ *is dynamically adjusted to achieve a constant power injection* Q_i*. More generally, such models include induction motors, load tap changers, and thermostatically controlled loads [33]. The local minimum of the energy function is clearly locally exponentially stable for an assortment of associated load dynamics, such*

as gradient $\beta \dot{V}_L = -\nabla E(V_L)$*, or damped second-order dynamics* $\alpha \ddot{V}_L = -\beta \dot{V}_L - \nabla E(V_L)$ *[39]. Static voltage stability is also relevant for slowly changing load profiles, where the fast stable load dynamics adiabatically track the network operating point (should it exist). Indeed, as Pal notes in [24],* "For loads with slow dynamics, the stability limit will occur at the same point as the maximum power *[transmission capacity]* determined from a power flow analysis." *For more general dynamic load models, our results can be interpreted as necessary for local dynamic stability, as the existence of a network operating point is an obvious prerequisite [40].* □

Supplementary Note 5 – Voltage Stability in Complex Power Networks

Single Load Example

To build intuition for our analysis in the case of complex networks, we present the results for the classic problem of a single generator feeding a single constant power load. For $n = 1$, the RPFE (8) is a single quadratic equation, and the necessary and sufficient condition for the existence of a solution follows immediately [33, Section 2.2.3].

Proposition 1 (Condition for Single Load)**.** *Consider the RPFE* (8) *for a single load (n=1)*

$$0 = q + bv(v - v^*)\,, \tag{20}$$

where $b < 0$*,* $v^* > 0$*, and let* $q_{\rm crit} \triangleq \frac{1}{4}b(v^*)^2 < 0$*. The following statements are equivalent:*

(i) **Small Loading:** $\Delta = q/q_{\rm crit} < 1$*;*

(ii) **High-Voltage Solution:** *There exists a unique voltage-stable solution* v^+ *to the RPFE* (20) *such that*

$$\frac{|v^+ - v^*|}{v^*} = \delta_- < \frac{1}{2}; \tag{21}$$

(ii) **Low-Voltage Solution:** *There exists a unique voltage-unstable solution* v^- *to the RPFE* (20) *such that*

$$\frac{|v_- - v^*|}{v^*} = \delta_+ > \frac{1}{2}\,. \tag{22}$$

Moreover, if any of the above statements are true, then δ_-, δ_+*, and* Δ *are related by*

$$\delta_\pm = \left(1 \pm \sqrt{1-\Delta}\right)/2\,. \tag{23}$$

Proposition 1 shows that a stable high-voltage solution exists only for loads which are less inductive than the critical inductive load $q_{\rm crit}$. Graphically, the situation is illustrated in Supplementary Fig. 2. Note that even in this simple scenario, the solution space of the RPFE is multi-valued. When $q = q_{\rm crit}$, the solutions v^+ and v^- coalesce at $v^*/2$ and vanish via saddle-node bifurcation [25]. In this simple one-dimensional case, we can derive a parametric formula for the Venikov index, given by [41, 42, 18]

$$k_{\rm V} \triangleq \frac{v^+ - v^*/2}{v^*/2} = \sqrt{1 - |\Delta|}\,. \tag{24}$$

As one may deduce from examining Supplementary Fig. 2, the Venikov index $k_{\rm V}$ measures the distance between the high-voltage solution v_L^+ and the low-voltage solution v_L^-, and thus gives an analytic measure of proximity to collapse.

Proof of Main Result for Complex Networks

The following simple necessary condition for solvability of (8) is inspired by the observations from Proposition 1.

Proposition 2 (Necessary Feasibility Condition). *Consider the decoupled reactive power flow equation* (8)*, and define the critical load matrix* $Q_{\rm crit} \in \mathbb{R}^{n\times n}$ *by*

$$Q_{\rm crit} \triangleq \frac{1}{4}[V_L^*]B_{LL}[V_L^*]\,. \tag{25}$$

If a solution to (8) *exists, then*

$$\frac{\mathbb{1}_n^T Q_L}{\mathbb{1}_n^T Q_{\rm crit}\mathbb{1}_n} \leq 1\,. \tag{26}$$

Proof: Defining a new variable $z \triangleq V_L - \frac{1}{2}V_L^*$ and substituting for V_L in (8), we obtain the equivalent reformulation

$$Q_L = Q_{\rm crit}\mathbb{1}_n - \frac{1}{2}[V_L^*]B_{LL}z + \frac{1}{2}[z]B_{LL}V_L^* - [z]B_{LL}z\,. \tag{27}$$

A necessary condition for the power flow equation(27) to hold true is that the sum over all equations holds true. Performing the sum (equivalently, left-multiplying by $\mathbb{1}_n^T$), the cross terms cancel and we obtain

$$\mathbb{1}_n^T Q_L = \mathbb{1}_n^T Q_{\rm crit}\mathbb{1}_n - z^T B_{LL} z\,. \tag{28}$$

Since $-B_{LL}$ is a symmetic nonsingular M-matrix (Assumption 1), B_{LL} is negative definite. Hence $z^T B_{LL} z \leq 0$ for all $z \in \mathbb{R}^n$ and the result follows. □

We refer to [11] for an alternative proof of Proposition 2. We note that the necessary voltage stability condition (26) is also *tight*, as it holds with equality when $Q_L = Q_{\rm crit}\mathbb{1}_n$, and a comparison with Proposition 1 shows that (26) is necessary and sufficient for a single load. Geometrically, the necessary condition of Proposition 2 restricts the vector of loads Q_L to a half-space. A downside of this necessary condition is that it provides only an aggregate bound on the load, and does not take into account how the load is distributed throughout the network in relation to voltage-regulated points, shunt capacitors, and so forth.

The following closed subset of voltage-space will help us quantify the area where desirable solutions to (8) should exist. Using the open-circuit voltages V_L^* defined in (9), for $\delta \in [0,1]$, define the compact, convex and partially-ordered *stability set* by

$$\overline{\mathcal{S}}(\delta) \triangleq \{V_L \in \mathbb{R}^n_{\geq 0} \mid (1-\delta)V_L^* \leq V_L \leq (1+\delta)V_L^*\}\,, \tag{29}$$

with $\mathcal{S}(\delta)$ being its interior. If $V_L \in \overline{\mathcal{S}}(\delta)$, then $|V_i - V_i^*|/V_i^* \leq \delta$ for each component i, and thus δ is simply a percentage deviation from the open-circuit voltage level.

Remark 2 (The Stability set & The Per-Unit System)**.** *From the definition of our stability set in* (29)*, the reader may be tempted to draw a one-to-one correspondence between the scaled voltages* V_i/V_i^* *(*$i \in \mathcal{L}$*) and the classic per-unit measurement system used by power engineers, where all voltage values in the network are scaled by uniform base voltage* V_i/V_{base}*. We strongly caution against drawing this equivalence. While the base voltage* V_{base} *is constant and uniform, the open-circuit voltages* V_i^* *defined in* (9) *vary bus-to-bus, and take into account non-uniform generator voltages, network topology, shunt compensation, constant current demands, and active power transfers through the power angles embedded in the effective susceptances* B_{ij}*. The discrepancy between* V_i/V_{base} *and* V_i/V_i^* *can be quite extreme in heavily shunt-compensated networks, and it has been frequently noted in the literature that per unit voltages are poor indicators of proximity to voltage collapse. Our results to follow suggest that the ratios* V_i/V_i^* *of the voltage magnitudes to their open-circuit values are more appropriate indicators of voltage stability margins.*

Finally, we note that that explicit upper and lower bounds $V^{\mathrm{lower}} \leq V_L \leq V^{\mathrm{upper}}$ *on the nodal voltage magnitudes may be present due to operational constraints. In general however, these upper and lower bounds may be asymmetric around the open circuit voltages, while the security set* (29) *is defined symmetrically. To conservatively formulate these bounds in terms of the stability set* (29)*, one may assume that* $V^{\mathrm{lower}} \leq V_L^* \leq V^{\mathrm{upper}}$ *and select* δ *as*

$$\delta_{\mathrm{low}}^{\mathrm{up}} = \min\left\{ -\max_{i\in\mathcal{L}}\left(\frac{V_i^{\mathrm{lower}}}{V_i^*} - 1\right), \min_{i\in\mathcal{L}}\left(\frac{V_i^{\mathrm{upper}}}{V_i^*} - 1\right)\right\} \in [0,1]\,. \tag{30}$$

With this choice, it holds that $\overline{\mathcal{S}}(\delta_{\mathrm{low}}^{\mathrm{up}}) \subseteq \{V_L \mid V^{\mathrm{lower}} \leq V_L \leq V^{\mathrm{upper}}\}$*, and thus any solutions which exist inside the set* $\overline{\mathcal{S}}(\delta_{\mathrm{low}}^{\mathrm{up}})$ *will also satisfy the explicit constraints.* □

The main result from the article is more formally stated as follows.

Supplementary Theorem 1 (Voltage Stability Condition for Complex Power Networks)**.** *For the RPFE* (8)*, define the stiffness matrix* $Q_{\mathrm{crit}} \in \mathbb{R}^{n\times n}$ *as in* (25)*. Assume that the network parameters and loads satisfy*

$$\Delta \triangleq \|Q_{\mathrm{crit}}^{-1}Q_L\|_\infty < 1\,, \tag{31}$$

and accordingly define the percentage deviations $\delta_- \in [0,\frac{1}{2}[$ *and* $\delta_+ \in]\frac{1}{2},1]$ *as the unique solutions to* $\Delta = 4\delta_\pm(1-\delta_\pm)$*. The following statements hold:*

1) **Secure Solution:** *There exists a unique locally voltage-stable solution* $V_L \in \overline{\mathcal{S}}(\delta_-)$ *of the RPFE* (8)*;*

2) **Solutionless Region:** *There exist no solutions of the RPFE* (8) *in the open set*

$$\{V_L \in \mathbb{R}^n \mid V_L > (1-\delta_+)V_L^* \text{ and } V_L \notin \overline{\mathcal{S}}(\delta_-)\}\,. \tag{32}$$

Moreover, the condition (31) *is tight: there exists a critical load profile* $Q_L = Q_{\mathrm{crit}}\mathbb{1}_n$ *for which 1) there exists no locally voltage-stable solution* $V_L \in \overline{\mathcal{S}}(\delta_-)$ *for any* $\delta_- \in [0,\frac{1}{2}[$*, and 2) there exists a solution of the RPFE* (8) *in the open set* (32) *for any* $\delta_- \in [0,\frac{1}{2}[$ *and any* $\delta_+ \in]\frac{1}{2},1]$*.*

Proof: **Statements 1 and 2):** Assuming for the moment that no component of V_L is zero, we may rearrange the RPFE (8) to obtain

$$V_L = V_L^* - B_{LL}^{-1}[V_L]^{-1}Q_L\,. \tag{33}$$

Consider the bijective change of variable $x \triangleq [V_L^*]^{-1}V_L - \mathbb{1}_n$. The new variable x can be thought of as a percentage deviation of V_L from the open-circuit voltage V_L^*. Note that from the definition of the stability set in (29), $V_L \in \overline{\mathcal{S}}(\delta)$ (resp. $\mathcal{S}(\delta)$) if and only if $x \in \overline{\mathcal{B}}_\infty(\delta)$ (resp. $\mathcal{B}_\infty(\delta)$). Writing (33) in terms of the new percentage deviation variable x, we obtain the equivalent representation

$$x = f(x) \triangleq -[V_L^*]^{-1}B_{LL}^{-1}[V_L^*]^{-1}[Q_L]r(x) \tag{34a}$$

$$= -\frac{1}{4}Q_{\mathrm{crit}}^{-1}[Q_L]r(x)\,, \tag{34b}$$

where Q_{crit} is as in (25) and $r(x) \triangleq (\frac{1}{1+x_1}, \ldots, \frac{1}{1+x_n})$. Having transformed the RPFE (8) into (34b), we now apply contraction mapping arguments to (34b). This procedure consists of two steps:

> **Step 1:** First, we regard (34b) as the equilibrium equation of the discrete-time dynamical system
>
> $$x(k+1) = f(x(k))\,, \qquad k \in \{1, 2, \ldots\}\,, \tag{35}$$
>
> and, under the parametric condition (31), show that there exists a $\delta \in [0, 1[$ such that the ∞-norm ball $\overline{\mathcal{B}}_\infty(\delta)$ is forward-invariant for the dynamics (35). In particular, we will show that there exists a $\delta_- \in [0, \frac{1}{2}[$ and a $\delta_+ \in]\frac{1}{2}, 1]$ such that $\overline{\mathcal{B}}_\infty(\delta)$ is forward-invariant for each $\delta \in [\delta_-, \delta_+]$, and that the iterates of (35) originating in $]-\delta_+, \infty[^n$ eventually reach the forward-invariant set $\overline{\mathcal{B}}_\infty(\delta_-)$.
>
> **Step 2:** Second, we show that f is a contraction mapping on the forward-invariant set $\overline{\mathcal{B}}_\infty(\delta_-)$, and apply the Banach Fixed Point Theorem [38, Chapter 9].

Step 1: Suppose $x(k) \in \mathcal{B}_\infty(1)$ for some $k \in \{1, 2, \ldots\}$. Then there exists a $\delta \in [0, 1[$ such that $x(k) \in \overline{\mathcal{B}}_\infty(\delta)$, with $\|x(k)\|_\infty = \delta$. We will first bound $\|x(k+1)\|_\infty$, and look for a condition under which $\|x(k+1)\|_\infty \leq \|x(k)\|_\infty$. We compute using (35) and (34b) that

$$\|x(k+1)\|_\infty = \frac{1}{4}\|Q_{\mathrm{crit}}^{-1}[Q_L]r(x(k))\|_\infty\,. \tag{36}$$

Using Assumptions 1 and 2 and Lemma 1, it holds that $-Q_{\mathrm{crit}}^{-1} = -4[V_L^*]^{-1}B_{LL}^{-1}[V_L^*]^{-1}$ has positive elements. Moreover, Q_L has nonpositive elements, and each component of $r(x(k))$ is strictly positive as $\|x(k)\|_\infty = \delta < 1$. We therefore compute

$$\frac{1}{4}\|Q_{\mathrm{crit}}^{-1}[Q_L]r(x(k))\|_\infty \leq \frac{\|Q_{\mathrm{crit}}^{-1}[Q_L]\|_\infty}{4}\|r(x(k))\|_\infty \tag{37a}$$

$$= \frac{\|Q_{\mathrm{crit}}^{-1}[Q_L]\|_\infty}{4}\|r(x(k))\|_\infty \tag{37b}$$

$$= \frac{\Delta}{4}\frac{1}{1-\delta}\,. \tag{37c}$$

Using this result and the fact that $\|x(k)\|_\infty = \delta$, it follows that $\|x(k+1)\|_\infty \leq \|x(k)\|_\infty$ if $\frac{\Delta}{4}\frac{1}{1-\delta} \leq \delta$, or equivalently

$$\Delta \leq 4\delta(1-\delta)\,. \tag{38}$$

The right-hand side of (38) is a nonnegative and concave function of $\delta \in [0,1[$, which achieves its global maximum of one at $\delta^* = \frac{1}{2}$. Thus, there exists a set of values for $\delta \in [0,1[$ with non-empty interior satisfying the inequality (38) if and only if (38) is true with strict inequality sign when $\delta = \delta^* = \frac{1}{2}$. This corresponds exactly to the parametric condition (31). If these equivalent conditions are true, there exist two unique values $\delta_- \in [0, \frac{1}{2}[$ and $\delta_+ \in [\frac{1}{2}, 1]$ satisfying (38) with equality sign, given by $\delta_\pm = \frac{1}{2}(1 \pm \sqrt{1-\Delta})$. These arguments are shown graphically in Supplementary Fig. 3. The preceding calculations show that for each $\delta \in [\delta_-, \delta_+]$, the set $\overline{\mathcal{B}}_\infty(\delta)$ is forward-invariant for the discrete-time dynamics (35), since $x(k) \in \overline{\mathcal{B}}_\infty(\delta)$ leads to $x(k+1) \in \overline{\mathcal{B}}_\infty(\delta)$. Moreover, for each $\delta \in [\delta_-, \delta_+]$ (resp. $\delta \in]\delta_-, \delta_+[$), we have that $\|x(k+1)\|_\infty \leq \|x(k)\|_\infty$ (resp. $\|x(k+1)\|_\infty < \|x(k)\|_\infty$) if $\|x(k)\|_\infty = \delta$. That is, the norm of iterates is non-increasing (resp. strictly decreasing). It follows that iterates of (35) originating in $\mathcal{B}_\infty(\delta_+)$ eventually reach the forward-invariant set $\overline{\mathcal{B}}_\infty(\delta_-)$.

To complete this step, note that since every component $r_i(x_i) = 1/(1+x_i)$ of $r(x)$ is a monotone decreasing function of x_i, for $x(k) \in]-\delta_+, +\infty[^n$, it follows that $\|r(x(k))\|_\infty < 1/(1-\delta_+)$, and hence, by the previous result, that $\|x(k+1)\|_\infty < \frac{\Delta}{4}\frac{1}{1-\delta_+} = \delta_+$; that is, we have $x(k+1) \in \mathcal{B}_\infty(\delta_+)$. Since f is continuous on $]-\delta_+, +\infty[^n$, it follows by combining the above results that all iterates of (35) originating in $]-\delta_+, \infty[^n$ reach $\overline{\mathcal{B}}_\infty(\delta_-)$. The discrete-time dynamics (35) therefore have no equilibria within the set $]-\delta_+, \infty[^n \setminus \overline{\mathcal{B}}_\infty(\delta_-)$, and thus (34b) has no solutions within the same set. This completes the proof of statement 2).

Step 2: Let $\delta \in [0, \delta_-]$, and let $x \in \overline{\mathcal{B}}_\infty(\delta)$. Using (34b), we calculate the Jacobian of f to be

$$\frac{\partial f}{\partial x}(x) = \frac{1}{4}Q_{\mathrm{crit}}^{-1}[Q_L][r(x)]^2 \tag{39}$$

and as before, bound it for $x \in \overline{\mathcal{B}}_\infty(\delta)$ as

$$\left\|\frac{\partial f}{\partial x}(x)\right\|_\infty \leq \frac{\Delta}{4}\left\|[r(x)]^2\right\|_\infty \leq \frac{\Delta}{4}\frac{1}{(1-\delta)^2}\,. \tag{40}$$

The map f is a contraction mapping on $\overline{\mathcal{B}}_\infty(\delta)$ if we have

$$\frac{\Delta}{4}\frac{1}{(1-\delta)^2} \leq \alpha \tag{41}$$

for some $\alpha \in [0,1[$ called the contraction rate. Selecting $\alpha \triangleq \delta/(1-\delta) < 1$, the contraction condition (41) is quickly seen to be equivalent to (38). We therefore once again have a set of values for δ such that f is a contraction map if and only if (31) holds, with δ_- being the limiting case of strict equality, yielding the contraction rate $\alpha = \delta_-/(1-\delta_-) < 1$. Thus, f is a contraction mapping on the invariant set $\overline{\mathcal{B}}_\infty(\delta_-)$. It now follows from the Banach Fixed-Point Theorem [38] that f has a unique fixed-point $x^* \in \overline{\mathcal{B}}_\infty(\delta_-)$, and therefore that the power flow (8) has a unique solution $V_L \in \overline{\mathcal{S}}(\delta_-)$.

To complete the proof of statement 1), it remains only to show that the unique solution $V_L \in \overline{\mathcal{S}}(\delta_-)$

is locally voltage-stable. From Lemma 2 this occurs if and only if the Hessian $H(V_L)$ in (17b) of the energy function (12) is positive definite. Inserting (33) into the second term of $H(V_L)$, we find that

$$H(V_L) = -B_{LL} + [V_L]^{-2}[Q_L]\,. \tag{42}$$

Substituting $V_L = [V_L^*](\mathbb{1}_n + x)$, left and right-multiplying by $[V_L^*]/2$ and simplifying using (25), we obtain

$$M_1 = \frac{1}{4}[V_L^*]H(V_L)[V_L^*] = -\frac{1}{4}[V_L^*]B_{LL}[V_L^*] + \frac{1}{4}[Q_L][\mathbb{1}_n + x]^{-2} \tag{43a}$$

$$= -Q_{\mathrm{crit}} + \frac{1}{4}[Q_L][\mathbb{1}_n + x]^{-2}\,. \tag{43b}$$

According to *Sylvester's Inertia Theorem* [43], M_1 has the same number of positive eigenvalues as $M_2 \triangleq -Q_{\mathrm{crit}}^{-1}M_1$, and thus M_1 is positive definite if and only if

$$I_n - \frac{1}{4}Q_{\mathrm{crit}}^{-1}[Q_L][\mathbb{1}_n + x]^{-2} \tag{44}$$

is anti-Hurwitz. Since $-Q_{\mathrm{crit}}$ is an irreducible M-matrix, the inverse Q_{crit}^{-1} has strictly negative elements. As it also holds that $Q_L \leq \mathbb{0}_n$, we conclude that $\frac{1}{4}Q_{\mathrm{crit}}^{-1}[Q_L][\mathbb{1}_n + x]^{-2}$ is a nonnegative matrix. The Gershgorin Circle Theorem [37] then implies that all eigenvalues of $\frac{1}{4}Q_{\mathrm{crit}}^{-1}[Q_L][\mathbb{1}_n + x]^{-2}$ are contained within a disc centered at the origin of radius equal to the maximum row sum of the matrix, or equivalently, its ∞-norm. We compute that

$$\frac{1}{4}\|Q_{\mathrm{crit}}^{-1}[Q_L][\mathbb{1}_n + x]^{-2}\|_\infty \leq \frac{\Delta}{4}\frac{1}{(1-\delta_-)^2} \leq \alpha < 1\,. \tag{45}$$

It follows that all eigenvalues of $\frac{1}{4}Q_{\mathrm{crit}}^{-1}[Q_L][\mathbb{1}_n + x]^{-2}$ are less than one, which shows the desired result.

Tightness: To show the moreover statement we proceed by contraposition, and construct a load profile Q_L for which $\Delta \geq 1$ and statements 1) and 2) fail. Consider the family of load profiles parameterized by $\alpha \in [0, 1]$ defined by $Q_L(\alpha) = \alpha \cdot Q_{\mathrm{crit}}\mathbb{1}_n$. Using (25) and (9) we compute that that

$$Q_{\mathrm{crit}}\mathbb{1}_n = \frac{1}{4}[V_L^*]B_{LL}[V_L^*]\mathbb{1}_n = \frac{1}{4}[V_L^*]B_{LL}V_L^* \tag{46a}$$

$$= -\frac{1}{4}[V_L^*]B_{LL}B_{LL}^{-1}(B_{LG}V_G + I_{\mathrm{shunt}}) \tag{46b}$$

$$= -\frac{1}{4}[V_L^*](B_{LG}V_G + I_{\mathrm{shunt}})\,. \tag{46c}$$

From Assumption 4 and Lemma 1, we therefore have that $Q_{\mathrm{crit}}\mathbb{1}_n \leq \mathbb{0}_n$. Hence, for every $\alpha \in [0, 1]$, $Q_L(\alpha) \leq \mathbb{0}_n$, and (31) yields $\Delta = \|Q_{\mathrm{crit}}^{-1}Q_L(\alpha)\|_\infty = \|\alpha Q_{\mathrm{crit}}^{-1}Q_{\mathrm{crit}}\mathbb{1}_n\|_\infty = \alpha\|\mathbb{1}_n\|_\infty = \alpha$.

Defining $\delta_\pm(\alpha) \triangleq \frac{1}{2}(1 \pm \sqrt{1-\alpha})$, one may verify by direct substitution that $V_L^+(\alpha) = (1 - \delta_-(\alpha))V_L^*$ and $V_L^-(\alpha) = (1 - \delta_+(\alpha))V_L^*$ are both particular solutions of (8). Moreover, for each $\alpha \in [0, 1[$, it holds that $V_L^+(\alpha)$ is the unique locally voltage-stable solution, located exactly at the vertex of $\overline{\mathcal{S}}(\delta_-(\alpha))$ closest to the origin, while V_L^- can be verified to be voltage-unstable, similarly located

at the vertex of $\overline{\mathcal{S}}(\delta_+(\alpha))$ closest to the origin. It follows that for $\alpha \in [0, 1[$, $V_L^+(\alpha)$ is the unique solution in $\overline{\mathcal{S}}(1/2)$, and that for $\alpha = 1$, $V_L^+(\alpha) = V_L^-(\alpha) = V_L^*/2$ and the two solutions coalesce at a point of saddle-node bifurcation [25]. By continuity, we therefore have that no solutions can cross the boundary of $\overline{\mathcal{S}}(1/2)$ from exterior to interior (that is, from $\mathbb{R}^n \setminus \overline{\mathcal{S}}(1/2)$ to $\mathcal{S}(1/2)$) as $\alpha \to 1$. Said differently, no previously existing solutions now belong to $\mathcal{S}(1/2)$. The only remaining option is that at $\alpha = 1$, a solution appears in $\mathcal{S}(1/2)$ via a codimension one bifurcation [25]. However, the sudden appearance of such a solution precludes the existence of a continuously differentiable function $G : \mathbb{R} \to \mathbb{R}^n$ defined on an open interval $\mathcal{A}$ of $\alpha = 1$ such $G(\alpha)$ solves the RPFE (8) with load profile $Q_L(\alpha)$ for each $\alpha \in \mathcal{A}$. It follows from the *Implicit Function Theorem* that the RPFE Jacobian (11) evaluated at this solution is singular [38]. Lemma 2 then precludes this new solution from being locally voltage-stable. Thus, there exists no value $\delta \in [0, \frac{1}{2}[$ such that a unique, locally voltage-stable solution exists in $\overline{\mathcal{S}}(\delta)$. This shows that statement 1) fails. To show that statement 2) fails as well, proceed by contradiction and assume that it holds. Then we expect there to exist values $\delta_- \in [0, \frac{1}{2}[$ and $\delta_+ \in]\frac{1}{2}, 1]$ such that the RPFE (8) possesses no solutions in the open set of (32). However, inspection shows that for any such values δ_- and δ_+, $V_L = V_L^*/2$ belongs to this set. This is a contradiction, completing the proof of the converse. □

Remark 3 (Interpretations of Supplementary Theorem 1)**.** *Supplementary Theorem 1 generalizes the one-dimensional result of Proposition 1 and Supplementary Fig. 2 to complex networks by taking into account the coupling between nodes of the network. Under the equivalent conditions of Supplementary Theorem 1, the positive orthant in the space of voltages is partitioned into three disjoint sets: the stability set* $\overline{\mathcal{S}}(\delta_-)$ *where the locally voltage-stable solution exists, a region surrounding the stability set where all solutions are forbidden, and a low-voltage regime which may or may not contain additional power flow equilibria. For the case of two loads, this partitioning is shown in Supplementary Fig. 4(b), with the corresponding partitioning of parameter-space shown in Supplementary Fig. 4(a). The inverse* Q_{crit}^{-1} *can be interpreted as the sensitivity matrix relating variations in load to variations in nodal voltage deviation (see* (50) *in "Power Network Perspective" below). The eigenvector corresponding to the largest eigenvalue of* $-Q_{\mathrm{crit}}^{-1}$ *determines the most sensitive directions in load-space [44, 45, 46]. An interesting contrast to the seminal works [44, 45, 46] is that* Q_{crit}^{-1} *is symmetric, indicating that the same eigenvector describes both the most sensitive direction in load-space as well as the most sensitive direction in scaled* (V_i/V_i^*) *voltage-space.* □

Remark 4 (Necessity of Stability Condition)**.** *While in general only a sufficient condition for the existence of a high-voltage solution, the condition* (31) *is tight for the critical loading profile* $Q_L = Q_{\mathrm{crit}}\mathbb{1}_n$*, for which* $\Delta = 1$ *and* $\delta_- = \delta_+ = 1/2$*, in agreement with the necessary and tight condition of Proposition 2, that is, for this direction in load space, the condition is both necessary and sufficient. To understand what this loading profile looks like, consider* (46c) *with* $I_{\mathrm{shunt}} = \mathbb{0}_n$ *for simplicity. Then the ith element of* $Q_{\mathrm{crit}}\mathbb{1}_n$ *is nonzero if and only if load bus* $i \in \mathcal{L}$ *is attached directly to a generator. Thus, this critical load profile corresponds to loading only at the most well-supported buses in the network, the load buses adjacent to generators.* □

Complex Networks Interpretation: Defining $V_{\min} \triangleq \min_{i\in\mathcal{L}}(V_L^*)_i$, a sufficient condition for (31) is that

$$\|Q_L\|_\infty < \frac{1}{4}|\lambda_1(B_{LL})|V_{\min}^2\,, \tag{47}$$

where $|\lambda_1(B_{LL})|$ is the smallest eigenvalue (in magnitude) of B_{LL}. This spectral condition, similar to the ones in [6, 7], uses only the first eigenvalue of the admittance matrix B_{LL} as a measure of network connectivity, while our more precise condition (31) implicitly uses all n network eigenvalues.

Power Network Perspective: In power system engineering, the nonlinear active power flow equations (3a) are often approximated by a heuristic called the "DC" power flow [26]. In the DC power flow, phase angles $\theta_i - \theta_j$ are assumed to be sufficiently small such that $\sin(\theta_i - \theta_j) \simeq \theta_i - \theta_j$, and voltage magnitudes V_i are approximated as $V_i = 1$ p.u. The active power balance (3a) then becomes a linear equation relating active power injections to phase angle differences. We now define a voltage/reactive power counterpart to this DC power flow approximation, and explain how its solution can be used to interpret the nonlinear voltage stability condition (31). Linearizing the right-hand side of (8) around the open-circuit solution V_L^*, we obtain [22, 7]

$$Q_L = -\mathrm{diag}(V_L^*) B_{LL} (V_{\mathrm{approx}} - V_L^*)\,. \tag{48}$$

Performing the change of variables to the percentage deviation vector x_{approx} via

$$V_{\mathrm{approx}} = \mathrm{diag}(V_L^*)(\mathbb{1}_n + x_{\mathrm{approx}}) \tag{49}$$

and solving, we obtain the solution

$$x_{\mathrm{approx}} = -\frac{1}{4} Q_{\mathrm{crit}}^{-1} Q_L\,. \tag{50}$$

Hence, the voltage stability condition (31) reads simply as $\|x_{\mathrm{approx}}\|_\infty < 1/4$, and can be interpreted as follows: the nonlinear power flow equations (8) have a unique, stable solution satisfying $|V_i - V_i^*|/V_i^* < 1/2$ if the percentage deviation *calculated from the linear power flow* (48) is less than 25% at each bus.

Resistive Circuit Interpretation: The voltage stability condition (31) can be interpreted as a restriction on the solution of a linear resistive circuit, defined on a new graph. Consider the original graph $G(\mathcal{V}, \mathcal{E})$ and construct a new graph $G_L(\mathcal{L}, \mathcal{E}_L)$ by 1) removing all generator buses $\mathcal{G}$, and 2) removing each weighted edge $\{i, j\} \in \mathcal{E}$ between generator i and load j, and replacing it with a shunt connection to ground of equal weight at node j; see Supplementary Fig. 1. For the nodal current injections $I \triangleq \mathrm{diag}(V_L^*)^{-1} Q_L$, and letting $v = (v_1, \ldots, v_n)$ be the vector of nodal voltages, the linear current balance relations are [14]

$$I = -B_{LL} v \qquad \Longleftrightarrow \qquad v = RI\,, \tag{51}$$

where $R \triangleq -B_{LL}^{-1}$ is the resistance matrix. Our voltage stability condition (31) then reads that $\|\mathrm{diag}(V_L^*)^{-1} RI\|_\infty = \|\mathrm{diag}(V_L^*)^{-1} v\|_\infty \leq \delta(1-\delta)$. That is, the normalized solution of this linear resistive circuit must have no node with voltage greater than $\delta(1-\delta)$. The elements $R_{ij} \geq 0$ of the resistance matrix quantify the *resistive distance* between load nodes, accounting for all current-carrying paths through the original circuit. The "driving point impedances" R_{ii} satisfy $R_{ii} \geq R_{ij}$ for all $i, j \in \mathcal{L}$ [4], and for distinct nodes $i, j \in \mathcal{L}$, $R_{ij} > 0$ if and only if there exists a path between i and j in the new graph $G_L(\mathcal{L}, \mathcal{E}_L)$. It follows from (51) that the voltage at node $i \in \mathcal{L}$ is most sensitive to changes in its local load demand Q_i, and decreasingly sensitive to load nodes that are more electrically distant. This captures the classic power systems intuition that reactive power

flows are *localized* in a network.

Supplementary Theorem 1 implies a useful voltage-space proximity index, which the reader may have inferred from Supplementary Fig. 4.

Corollary 1 (Proximity to Low-Voltage Solution)**.** *Define the parametric Venikov index*

$$K_{\mathrm{V}} \triangleq \sqrt{1-\Delta}\,, \tag{52}$$

and let $V_L^{\mathrm{other}} \in \mathbb{R}^n$ be any solution of the RPFE (8) *other than the secure solution of Supplementary Theorem 1 statement 1). Then*

$$\frac{|V_i - V_i^{\mathrm{other}}|}{V_i^*} \geq K_{\mathrm{V}}\,, \quad i \in \mathcal{L}\,. \tag{53}$$

Proof: While from Supplementary Theorem 1 statement 1) the secure solution belongs to the set $\overline{\mathcal{S}}(\delta_-)$, from Supplementary Theorem 1 statement 2) we have that all other solutions must lie outside the set $](1-\delta_+)V_L^*, \infty_n[$, and therefore in particular outside the set $\mathcal{S}(\delta_+)$. We may therefore write $V_L = [V_L^*](\mathbb{1}_n + x)$ and $V_L^{\mathrm{other}} = [V_L^*](\mathbb{1}_n + x^{\mathrm{other}})$ for $x \in \overline{\mathcal{B}}_\infty(\delta_-)$ and $x^{\mathrm{other}} \in \mathbb{R}^n \setminus \mathcal{S}(\delta_+)$. We compute that

$$\|[V_L^*]^{-1}(V_L - V_L^{\mathrm{other}})\|_\infty = \|x - x^{\mathrm{other}}\|_\infty \geq \delta_+ - \delta_-\,. \tag{54}$$

From Supplementary Theorem 1 it holds that $\Delta = 4\delta_\pm(1-\delta_\pm)$, and therefore that $\delta_\pm = \frac{1}{2}(1 \pm \sqrt{1-\Delta})$. We therefore compute that

$$\delta_+ - \delta_- = \frac{1}{2}\left(1+\sqrt{1-\Delta}\right) - \frac{1}{2}\left(1-\sqrt{1-\Delta}\right) \tag{55a}$$

$$= \sqrt{1-\Delta} = K_{\mathrm{V}}\,, \tag{55b}$$

which completes the proof. □

By direct comparison, the index (52) is seen to be an appropriate multi-dimensional generalization of the parametric Venikov index (24). To the authors knowledge, this is the first such parametric result available in the literature. In terms of the energy function (12), $\sqrt{1-\Delta}$ can be understood as a lower bound on the distance between the stable and unstable equilibria; c.f. Figure 1(c) of the main article.

Remark 5 (The Effects of Power Angles and Decoupling)**.** *As can be seen by examining* (2b)*, the voltage phase angles $\theta_i - \theta_j$ enter the reactive power flow equations as a product $B_{ij}\cos(\theta_i - \theta_j)$ with the off-diagonal elements of the admittance matrix B. It follows that any deviation of the phase angle differences from zero tends to* weaken *the effective coupling term $B_{ij}\cos(\theta_i - \theta_j)$ between buses $i, j \in \mathcal{L}$. This is particularly clear for the two node system of* (20)*. In this case, one may verify that $v^* = V_G\cos(\theta)$, that $q_{\mathrm{crit}} = \frac{1}{4}bV_G^2\cos^2\theta$, and that $\Delta = q_L/q_{\mathrm{crit}}$, where θ is the angular difference between the two nodes. As $|\theta|$ increases, the reactive power limit q_{crit} decreases, and tends to zero as $|\theta| \to \pi/2$. Moreover, $1/q_{\mathrm{crit}}$ is exactly the sensitivity of the loading margin Δ with respect to changes in reactive power demands, and this sensitivity increases as active power flows increase. This is elegantly expressed by Van Cutsem in [47], where he writes "...the reactive power margin implicitly reflects the system stress imposed by the active power transfers, since the*

margin is a reserve after imposing the active load to be satisfied. As an example, if one would (fictitiously) approach collapse through only active load increases, the reactive margin would tend to zero as well."

In terms of the vector formulation (7)*, the above observations translate to a reduction in the off-diagonal elements of the sub-block* B_{LL} *as well as a reduction in the non-zero elements of* B_{GL}*. Accordingly, this active/reactive power coupling tends to weaken the stiffness matrix* Q_{crit}*, thereby increasing the network stress* Δ*. To summarize, increasing active power flows appear in our formulation through the stiffness matrix* Q_{crit}*, and lead to increasing sensitivity of voltage deviations with respect to changes in reactive power demands. While not a full description of the nonlinear cross-coupling between active and reactive power flow, this behavior is consistent with the knowledge that increasing active power demands compromise voltage stability and push the system towards voltage collapse [47, 18, 48].*

While active/reactive power coupling is mostly negligible in normal transmission system operation and under light loading conditions, it can become the dominant effect as voltage collapse is approached. There are two factors to consider here. Firstly, using any fixed set of phase angles in the effective susceptance matrix will underestimate the phase angles that occur as one moves closer to voltage collapse; the stiffness of the effective stiffness matrix Q_{crit} *will therefore be overestimated. The second factor is less important and more subtle: solvability of the coupled power flow equations is not equivalent to individual solvability of decoupled active and reactive power flow. We therefore expect the analytic stability condition derived for the simplified decoupled reactive power flow* (8) *to be optimistic near the point of collapse, unless power factors are low. Our voltage collapse simulation study in the main article (see Experiment 2 in Supplementary Methods for details) confirms this intuition.* □

Supplementary Note 6 – Monotonicity of Stability Margins With Respect to Parameters: Results and Counterexamples

We now briefly explore how perturbations in the network parameters influence the value of the loading margin Δ defined in (31). From the spring network analogy of Figure 1(b) in the main article and from general intuition, one would expect the following monotonicity results:

(a) strengthening the connection between any generator and any load should decrease the value of Δ;

(b) increasing generator voltage levels should decrease Δ;

(c) increasing shunt compensation should decrease Δ.

It turns out that statement (a) is false in general, while statements (b) and (c) are true.

Example 1 (Counterexample to (a))**.** *Consider two generators at voltages* V_1 *and* V_2 *connected to a single load at voltage* V_0 *through susceptances* $-b_1$ *and* $-b_2$*, with a shunt capacitor of susceptance* b_{s} *present at the load. Assumption 1 in this case becomes* $b_1 + b_2 - b_{\mathrm{s}} > 0$*, and we assume for simplicity that no constant current load is present at the load. Then the reactive power balance equation* (7) *becomes*

$$Q_L = V_0\left((b_1 + b_2 - b_{\mathrm{s}})V_0 - b_1V_1 - b_2V_2\right)\,. \tag{56}$$

One may verify explicitly that Q_{crit} in this case is given by

$$|Q_{\text{crit}}| = \frac{(V_1 b_1 + V_2 b_2)^2}{4(b_1 + b_2 - b_{\text{s}})}\,. \tag{57}$$

Since there is only a single load, monotonicity of $\Delta = Q_L/Q_{\text{crit}}$ with respect to changes in b_1 or b_2 is equivalent to monotonicity of Q_{crit}. In particular, conventional engineering wisdom suggests that $|Q_{\text{crit}}|$ should be an increasing function of either parameter, that is, strengthening the network should reduce the risk of collapse. An easy calculation gives that

$$\frac{\mathrm{d}}{\mathrm{d}b_2}|Q_{\text{crit}}| = \frac{V_2}{4}\frac{(V_1 b_1 + V_2 b_2)}{b_1 + b_2 - b_{\text{s}}}\left(b_2 - 2b_{\text{s}} + 2b_1\left(1 - \frac{1}{2}\frac{V_1}{V_2}\right)\right)\,. \tag{58}$$

When $b_2 > 2b_{\text{bs}} - 2b_1(1 - V_1/2V_2)$, $|Q_{\text{crit}}|$ is a monotone increasing function of b_2 as expected. When this inequality is violated however we find that strengthening the second transmission line decreases *stability margins. In the case $b_{\text{s}} = 0$, this turning point will exist if and only if $V_1 > 2V_2$, while if $V_1 = V_2 = V$, this turning point exists if and only if $b_{\text{s}} > b_1/2$. While these parametric conditions are extreme and would not occur in real-world networks, this example highlights the theoretical issue with claim (a), and nonetheless suggests that one should be cautious when upgrading transmission infrastructure.* □

In this example we observe that either strong heterogeneity of generator voltages, the presence of large shunts, or both can cause a lack of monotonicity. We will show that when these factors are absent, the original intuition is correct. Consider a nominal network, and let $b_{LG} \in \mathbb{R}^{n\times m}_{\geq 0}$ be a nonnegative matrix where the ijth element is the desired increase in coupling between generator $i \in \mathcal{G}$ and load $j \in \mathcal{L}$. To avoid the trivial case where the original and modified networks are identical, we assume that at least one element of b_{LG} is strictly positive. The modified network is represented by the new network matrices $\overline{B}_{LG} = B_{LG} + b_{LG}$, and $\overline{B}_{LL} = B_{LL} - \text{diag}(b_{LG}\mathbb{1}_m)$, along with the new open-circuit voltages $\overline{V_L^*} = -\overline{B}_{LL}^{-1}\overline{B}_{LG}V_G$. For simplicity, we ignore constant current loads in the following proposition.

Proposition 3 (Effects of Load-Generator Coupling Increases)**.** *Consider the nominal network and the modified network as described above, with stiffness matrices $Q_{\text{crit}} = \frac{1}{4}[V_L^*]B_{LL}[V_L^*]$ and $\overline{Q}_{\text{crit}} = \frac{1}{4}[\overline{V_L^*}]\overline{B}_{LL}[\overline{V_L^*}]$ respectively. Assume that there are no shunt susceptances present, and that all generator voltages are equal to the same constant $V_g > 0$. Then it holds element-wise that $\overline{Q}_{\text{crit}}^{-1} > Q_{\text{crit}}^{-1}$.*

Since Q_{crit}^{-1} and $\overline{Q}_{\text{crit}}^{-1}$ are strictly negative matrices (Assumptions 1–2), the inequality $\overline{Q}_{\text{crit}}^{-1} > Q_{\text{crit}}^{-1}$ indicates that the elements of $\overline{Q}_{\text{crit}}^{-1}$ are smaller in magnitude than those of Q_{crit}^{-1}. It follows immediately that $\overline{\Delta} < \Delta$; the network stress has decreased. Comparing Proposition 3 to the results of Example 1, we note that the assumptions of no shunt susceptances and uniform generator voltages are only sufficient for monotonicity, and not necessary. Nonetheless, the parametric setting in Proposition 3 is much closer to reality than the exotic parameters required in Example 1.

Proof. Under the assuption of no shunt susceptances, the matrices $-B_{LL}^{-1}B_{LG}$ and $-\overline{B}_{LL}^{-1}\overline{B}_{LG}$ are both row-stochastic [49, Lemma II.1]. Since $V_G = V_g\mathbb{1}_m$, it follows that $V_L^* = \overline{V_L^*} = V_g\mathbb{1}_n$, and

hence that $Q_{\text{crit}} = V_g^2 B_{LL}/4$ and $\overline{Q}_{\text{crit}} = V_g^2 \overline{B}_{LL}/4$. By Assumptions 1–2, it holds that $-B_{LL}$ is an irreducible M-matrix, and since b_{LG} is nonnegative, it follows that $-\overline{B}_{LL} = -B_{LL} + \text{diag}(b_{LG}\mathbb{1}_m)$ is also an irreducible M-matrix. We conclude that $-\overline{B}_{LL} + B_{LL}$ is positive definite. It follows immediately by properties of irreducible M-matrices [50] that $-\overline{B}_{LL}^{-1} < -B_{LL}^{-1}$ element-wise, and the result follows. □

To study changes in generator voltages, we again consider a nominal network and a modified network, where now the modified network is constructed by changing the generator voltage setpoints to $\overline{V}_G = V_G + v_G$, where $v_G \geq \mathbb{0}_n$ with at least one strictly positive element. The open-circuit voltages in the modified network are given by $\overline{V_L^*} = -B_{LL}^{-1} B_{LG} \overline{V}_G$. In this case, we observe, as expected, that raising the generator voltages improves the distance to voltage collapse.

Proposition 4 (Effects of Generator Voltage Increases)**.** *Consider the nominal network and the modified network as described above, with stiffness matrices* $Q_{\text{crit}} = \frac{1}{4}[V_L^*] B_{LL} [V_L^*]$ *and* $\overline{Q}_{\text{crit}} = \frac{1}{4}[\overline{V_L^*}] B_{LL} [\overline{V_L^*}]$*. Then it holds element-wise that* $\overline{Q}_{\text{crit}}^{-1} > Q_{\text{crit}}^{-1}$.

Proof. Note that $\overline{V_L^*} = -B_{LL}^{-1} B_{LG} \overline{V}_G = -B_{LL}^{-1} B_{LG}(V_G + v_G) = V_L^* + v_L^*$, where $v_L^* = -B_{LL}^{-1} B_{LG} v_G$. From Assumptions 1–2, it follows that $-B_{LL}^{-1} B_{LG}$ is a positive matrix, consequently $v_L^* > \mathbb{0}_n$ component-wise, and hence that $\overline{V_L^*} > V_L^*$ component-wise as well. Note then that for any $i, j \in \mathcal{L}$,

$$-(\overline{Q}_{\text{crit}}^{-1})_{ij} = \frac{-4(B_{LL}^{-1})_{ij}}{\overline{V_i^*} \cdot \overline{V_j^*}} < \frac{-4(B_{LL}^{-1})_{ij}}{V_i^* \cdot V_j^*} = -(Q_{\text{crit}}^{-1})_{ij}\,, \tag{59}$$

which shows the desired result. □

Finally, to study changes in shunt compensation we again consider a nominal network and a modified network. We let $B_{\text{s}} = \text{diag}(b_{\text{s},1}, \ldots, b_{\text{s},n}) \geq \mathbb{0}_n$ denote the diagonal matrix of additional shunt capacitors affixed at the load nodes, and we assume that at least one element of B_{s} is strictly positive. The grounded susceptance matrix for the modified network is given by $\overline{B}_{LL} = B_{LL} + B_{\text{s}}$, which we assume is also a (negative) M-matrix. The modified open-circuit voltages are $\overline{V_L^*} = -\overline{B}_{LL}^{-1} B_{LG} V_G$. We omit the proof of the following result, which can be shown similarly to the previous two. The following result confirms the conventional practice that shunt compensation improves the stability of the network. We omit the proof, which follows analogous arguments as the proofs of Proposition 3 and 4.

Proposition 5 (Effects of Shunt Capacitor Increases)**.** *Consider the nominal network and the modified network as described above, with stiffness matrices* $Q_{\text{crit}} = \frac{1}{4}[V_L^*] B_{LL} [V_L^*]$ *and* $\overline{Q}_{\text{crit}} = \frac{1}{4}[\overline{V_L^*}] \overline{B}_{LL} [\overline{V_L^*}]$*. Then it holds element-wise that* $\overline{Q}_{\text{crit}}^{-1} > Q_{\text{crit}}^{-1}$.

In summary, we find that the stability margin Δ defined in (31) can be leveraged to provide insight into how the parameters of the original network influence voltage stability, and that the stability margin may display a lack of monotonicity with respect to parameters.

Supplementary Note 7 – Voltage Stability Condition Incorporating Generator Injection Limits

As discussed in the generator modeling of Supplementary Note 2, synchronous generators are typically subject to capability curve limits [18], which in the context of this paper correspond to upper and lower limits on the reactive power Q_i which can be injected at a generator $i \in \mathcal{G}$. While the results of Supplementary Theorem 1 (or equivalently, Theorem 1 of the main article) give sufficient conditions for the existence of a unique voltage-stable solution of (6), no *a priori* constraints were placed on the resulting generator reactive power injections, which are determined *a posteriori* by evaluating the right-hand side of (2b) at the system operating point. There may be situations where the physical network is able to support the transfer of reactive power (that is, (6) possess a voltage-stable solution), but the resulting generator limits are violated, meaning that the operating point is infeasible in practice. Our goal now is to generalize the voltage stability condition of Supplementary Theorem 1 to ensure the existence of a voltage-stable solution subject to generator reactive power injections satisfying predetermined limits.

The generator reactive power injections $Q_G \in \mathbb{R}^m$, given by (2b) at nodes $i \in \mathcal{G}$, are written in vector notation as

$$Q_G = -[V_G](B_{GG}V_G + B_{GL}V_L)\,, \tag{60}$$

where B_{GG} and B_{GL} are the appropriate blocks of the effective susceptance matrix (refer back to the block partitioned matrix (1)). Here we will focus on generator injection upper bounds $Q_G^{\text{upper}} \in \mathbb{R}^m$ (so-called overexcitation) since this is the most relevant case for voltage collapse; analogous arguments can be made for underexcitation limits. We introduce two quantities related to generator injections that will help us to succinctly state our results. We define the open-circuit generator injections $Q_G^* \in \mathbb{R}^m$ and the auxiliary injections $Q_{\text{aux}} \in \mathbb{R}^m_{\geq 0}$ by

$$Q_G^* \triangleq -[V_G](B_{GG}V_G + B_{GL}V_L^*)\,, \tag{61}$$

$$Q_{\text{aux}} \triangleq [V_G]B_{GL}V_L^*\,. \tag{62}$$

The open-circuit injections Q_G^* are the generator injections one would observe when the constant power loads Q_L are set to zero, while the vector of auxiliary injections Q_{aux} will serve as useful scaling factors. We make the plausible and necessary assumption that $Q_G^* < Q_G^{\text{upper}}$ component-wise; the open-circuit injections satisfy the generator limits. For convenience, let $\bar{\mathcal{G}} = \{i \in \mathcal{G} \mid \text{there exists } j \in \mathcal{L} \text{ s.t. } B_{ij} \neq 0\}$ be the set of generators which are connected to at least one load. It is straightforward to see that if $i \in \bar{\mathcal{G}}$ then the corresponding component of Q_{aux} as defined in (62) is non-zero.

The next result generalizes Supplementary Theorem 1 (and hence, Theorem 1 of the main article) to account for generator limits.

Supplementary Theorem 2 (Voltage Stability Condition Including Generator Limits)**.** *Consider the RPFE* (8) *and the generator injections* (60)*. Let $Q_G^{\text{upper}} \in \mathbb{R}^m$ be desired upper bounds in the generator injections, $Q_G < Q_G^{\text{upper}}$, and let the open-circuit injections Q_G^* and the auxiliary injections Q_{aux} be as defined as in* (61)*–*(62)*. Consider the (normalized) minimum generator injection slack*

$$\delta_{\text{constr}} \triangleq \min_{i \in \bar{\mathcal{G}}} \frac{Q_{G,i}^{\text{upper}} - Q_{G,i}^*}{Q_{\text{aux},i}} > 0\,, \tag{63}$$

and let $\overline{\Delta} > 0$ *be defined by*

$$\overline{\Delta} \triangleq \begin{cases} 4\delta_{\mathrm{constr}}(1-\delta_{\mathrm{constr}}), & \text{if } \delta_{\mathrm{constr}} < 1/2, \\ 1, & \text{if } \delta_{\mathrm{constr}} \geq 1/2. \end{cases} \tag{64}$$

Assume that

$$\Delta \triangleq \|Q_{\mathrm{crit}}^{-1} Q_L\|_\infty < \overline{\Delta}\,, \tag{65}$$

and accordingly define the percentage deviation $\delta_{\mathrm{unconstr}}$ *as the unique solution to* $\Delta = 4\delta(1-\delta)$ *belonging to the interval* $[0, \frac{1}{2}[$. *Finally, set* $\delta_- = \min(\delta_{\mathrm{constr}}, \delta_{\mathrm{unconstr}}) \in [0, \frac{1}{2}[$. *Then the RPFE (8) has a unique voltage-stable solution* $V_L \in \overline{\mathcal{S}}(\delta_-)$ *and the generator injections (60) satisfy the constraints* $Q_G < Q_G^{\mathrm{upper}}$.

Before proving Supplementary Theorem 2 we make several comments. First, note that $\overline{\Delta}$ depends only on δ_{constr}, which in turn depends only on fixed parameters of our problem setup, such as the network topology/weights, generator voltage setpoints, and generator injection limits. Thus, like the stability condition (31) in Supplementary Theorem 1, the stability condition (65) is purely parametric.

Second, to see that the unconstrained case of Supplementary Theorem 1 is embedded in Supplementary Theorem 2, consider what happens when the generator injections limits Q_G^{upper} are large. Then the generator injection slack δ_{constr} in (63) is large, $\overline{\Delta}$ in (64) evaluates to 1, and the stability condition (65) reduces to the previous condition of Supplementary Theorem 1. In this regime, the network is limited only by insolvability of the reactive power flow equations, and not by generator limits; the same argument holds whenever $\delta_{\mathrm{constr}} > \delta_{\mathrm{unconstr}}$. Conversely, when $\delta_{\mathrm{constr}} < \delta_{\mathrm{unconstr}}$, network stability is limited by generator limits, and $\overline{\Delta} - \Delta$ becomes the loading margin which implicitly quantifies the remaining slack in parameter-space for which generation limits are guaranteed to be met. The conservativeness of this stability margin will vary from network to network depending on the precise values of the generator limits Q_G^{upper}.

Proof: We first prove the statements regarding the unique solution of (8). First consider the case where $\delta_{\mathrm{constr}} \geq 1/2$. It then holds that $\overline{\Delta} = 1$, the condition (65) reduces to the unconstrained stability condition (31) of Supplementary Theorem 1, $\delta_- = \delta_{\mathrm{unconstr}} = (1 - \sqrt{1-\Delta})/2$, and all the conclusions follow. In the case where $\delta_{\mathrm{constr}} < 1/2$, the inequality (65) takes the form $\Delta \leq 4\delta_{\mathrm{constr}}(1-\delta_{\mathrm{constr}})$. Comparing this to (38) from the proof of Supplementary Theorem 1, it follows that there exists a unique voltage-stable solution $V_L \in \overline{\mathcal{S}}(\delta_{\mathrm{constr}})$ to the RPFE (8). Moreover, since $\Delta < \overline{\Delta} < 1$, $\delta_{\mathrm{unconstr}}$ also remains well defined, so there exists a unique voltage-stable solution $V_L \in \overline{\mathcal{S}}(\delta_{\mathrm{unconstr}})$ as well. It follows that there exists a unique voltage-stable solution $V_L \in \overline{\mathcal{S}}(\delta_{\mathrm{constr}}) \cap \overline{\mathcal{S}}(\delta_{\mathrm{unconstr}}) = \overline{\mathcal{S}}(\delta_-)$ as claimed.

It remains only to show that the generator constraints are satisfied. Under the assumed conditions we can write the load voltages as $V_L = [V_L^*](\mathbb{1}_n + x)$ where x belongs to the vector interval $x \in [-\delta_-\mathbb{1}_n, \delta_-\mathbb{1}_n]$. Using this and (61), substitution shows that the generator injections (60) can be written as

$$Q_G = -[V_G](B_{GG}V_G + B_{GL}V_L) = Q_G^* - [V_G]B_{GL}[V_L^*]x = Q_G^* - Q_{\mathrm{aux}}x\,. \tag{66}$$

Since $[V_G]B_{GL}[V_L^*]$ is a nonnegative matrix and $V_L \in \overline{\mathcal{S}}(\delta_-)$ (meaning that all $x_i \in [-\delta_-, \delta_-]$), we

may upper bound the right-hand side of (66) by inserting $-\delta_{-}\mathbb{1}_n$ for x, resulting in the element-wise vector inequality

$$Q_G \leq Q_G^* + \delta_{-} \cdot Q_{\text{aux}} \leq Q_G^* + \delta_{\text{constr}} \cdot Q_{\text{aux}}\,, \tag{67}$$

where the second inequality follows since $\delta_{\text{constr}} \geq \delta_{-}$ by definition. There are now two cases to consider. If $Q_{\text{aux},i} = 0$, then the corresponding constraint is satisfied since $Q_{G,i}^* < Q_{G,i}^{\text{upper}}$ by assumption. If $Q_{\text{aux},i} \neq 0$, then the corresponding constraint $Q_{G,i}^* < Q_{G,i}^{\text{upper}}$ is satisfied if and only if $\delta_{\text{constr}} \leq (Q_{G,i}^{\text{upper}} - Q_{G,i}^*)/Q_{\text{aux},i}$ at each generator $i \in \mathcal{G}$, which holds by the definition of δ_{constr}. Thus the generator constraints are satisfied, completing the proof. □

Supplementary Methods

Here we provide additional details regarding the numerical experiments presented in the main article.

Experiment 1 – Voltage Stability Assessment for Test Networks

To establish the accuracy of the voltage stability condition (31) over a large set of power networks, we consider a smart grid scenario in which both generation and demand are subject to fluctuations due to a high penetration of renewable generation and controllable demand response. To generate a large sample of randomized test cases, we modify the nominal simulation parameters [51, 52] as follows. For each of the eleven test cases under consideration, we construct 1000 realizations by

(i) **Fluctuating load:** Through a uniform distribution over all buses, we first select 30% of buses in the network for load randomization. The base case active/reactive power injections (P_i, Q_i) at each selected bus are randomized as $(1+\alpha_i)P_i$ and $(1+\alpha_i)Q_i$, where α_i is pulled from a Gaussian distribution with mean zero and standard deviation 0.5.

(ii) **Fluctuating generation:** Through a uniform distribution over all generator buses, we first select 30% of the generators in the network for randomization. The base case active power generation P_i at each selected generator is randomized as $(1+\alpha_i)P_i$, where α_i is pulled from a Gaussian distribution with mean zero and standard deviation 0.3.

(iii) **Redispatch:** Since the above randomization procedure can lead to a large imbalance between total scheduled generation and total demand, we calculate the imbalance (neglecting losses) and distribute the imbalance uniformly across the unrandomized generators.

For each realization, we numerically calculate using MATPOWER [51] the exact solution $(\theta, V_L) \in \mathbb{T}^{n+m} \times \mathbb{R}^n_{>0}$ to the lossless coupled power flow equations (3a) (if the Newton-Raphson solver diverges while attempting to find the solution, we discard the trial and repeat it). We then compute the maximum voltage deviation of the exact solution as

$$\delta_{\text{exact}} \triangleq \max_{i \in \mathcal{L}} \frac{|V_i - V_i^*|}{V_i^*}\,. \tag{68}$$

To apply our theory, we use the calculated phase angles θ to build the effective susceptance matrix (Supplementary Note 2, Power Angle Decoupling), we compute the open-circuit voltages (9), the stiffness matrix (25), compute Δ from (31) and finally compute our estimate $\delta_- = \frac{1}{2}(1 - \sqrt{1-\Delta})$ of the worst case normalized voltage deviation. The first column of Table 1 in the main article indicates that for all realizations constructed, it held that $\Delta < 1$ and that $\delta_{\rm exact} \leq \delta_-$ as expected from Supplementary Theorem 1. The second, third, and fourth columns of Table 1 list the average values obtained for $\delta_{\rm exact}$, δ_- and $(\delta_- - \delta_{\rm exact})/\delta_{\rm exact}$ over all realizations for each network.

Experiment 2 – Δ as an Indicator of Voltage Collapse

In this experiment the continuation power flow implemented in MATPOWER was used to drive the New England 39 bus system towards voltage collapse, and test whether and how well the metric Δ performs as a predictor of collapse. The direction in parameter space along which the system is incrementally loaded is specified in terms of a baseline set of loads and a target set of loads. The system is then loaded along the ray connecting the baseline configuration and the target configuration, tracing out the top-half of the nose curves shown in Figures 3 and 4 of the main article.

For our first study, the baseline profile was chosen as the default loading profile (P, Q_L). The average power factor for load buses in this baseline profile is 0.88. The target profile was constructed by modifying the baseline profile as $(P, 3.1 \times Q_L)$, where the numerical value of 3.1 was selected such that the average power factor of load buses in the target profile equaled 0.7. This corresponds to a heavily reactive target profile, and hence as the system is loaded from the base profile through the target profile, the power factors of loads decrease, as is often the case under stressed network conditions.

For our second study, the baseline profile was again chosen as the default loading profile, and the target profile was also chosen as the default loading profile. Thus, as the system was loaded towards voltage collapse, all power injections were simply scaled up proportionally, with loads maintaining their original power factors of 0.88 on average.

For both studies, V_L^* and Δ were computed as described in Experiment 1 for the lossless coupled power flow model (3a)–(3b). Bus four was selected for plotting purposes since it represented the most stressed bus in the network, displaying both the lowest per unit voltage along with having the highest component value from the vector $Q_{\rm crit}^{-1} Q_L$. This was therefore the bus at which our theoretical bound $V_i \geq V_i^*(1 - \delta_-)$ was most likely to be violated. As can be seen in Figures 3 and 4, the bound is quite tight near the base case and becomes looser as the system is progressively loaded.

Experiment 3 – Corrective Action

In this case study, we illustrate the utility of our condition (31) for online network stability monitoring and corrective action. First, we highlight how capacitive shunt compensation acts as a double-edged sword: capacitor banks assist in supporting the voltage magnitudes at load buses of the network, but simultaneously makes the true network stress unobservable through the network voltage profile. We show that our condition (31) can be used to accurately assess this hidden danger

by providing a quantitative estimate of network stress. Second, we show how the voltage stability condition (31) suggests corrective action schemes to increase the stability margin of the network.

The stressed network case was created as follows, beginning from the base case data for the NE 39 bus system from MATPOWER. First, the network contained two capacitive loads, which we flipped in sign to create a heavier inductive loading profile. Next, all power demands in the network were scaled up uniformly by 70%, with their original power factors maintained. To avoid unrealistically overloading the slack bus, the resulting imbalance was dispatched uniformly over all generators. Loading at buses seven through nine was increased an additional 50%, again maintaining the original power factors. The power factor of bus eight was then lowered to 0.82 by adding additional reactive load. Shunt compensation was placed uniformly across the network to bring most of the voltages back within operational limits, with additional shunt compensation being placed locally at buses seven through nine to compensate for the additional loading present. The power flow was then solved for the full coupled power flow model including branch conductances.

Control action was implemented by curtailing the reactive power demands at buses seven and nine in the manner described in the main article. Accompanying this curtailment was an *equal* reduction in shunt compensation across buses seven through nine. That is, the total MVAR which would have been injected by the removed capacitors (at 1 p.u.) equaled the total reactive power curtailment applied at buses seven and nine. This can also be seen from Figure 5; the per-unit voltage profiles before and after are nearly identical, but stability margins (plotted as V_i/V_i^*) are substantially improved by substituting stiff reactive power injections for shunt compensation, and properly selecting the locations for reactive power injection.

Supplementary References

[1] Meyer, C. & Stadelmaier, M. Singular M-matrices and inverse positivity. *Linear Algebra and its Applications* **22**, 139–156 (1978).

[2] Berman, A. & Plemmons, R. J. *Nonnegative Matrices in the Mathematical Sciences* (SIAM, 1994).

[3] Wu, F. F. & Kumagai, S. *Limits on Power Injections for Power Flow Equations to Have Secure Solutions* (Electronics Research Laboratory, College of Engineering, University of California, 1980).

[4] Wu, F. & Kumagai, S. Steady-state security regions of power systems. *IEEE Transactions on Circuits and Systems* **29**, 703–711 (1982).

[5] Kaye, R. & Wu, F. Analysis of linearized decoupled power flow approximations for steady-state security assessment. *IEEE Transactions on Circuits and Systems* **31**, 623–636 (1984).

[6] Thorp, J., Schulz, D. & Ilić-Spong, M. Reactive power-voltage problem: conditions for the existence of solution and localized disturbance propagation. *International Journal of Electrical Power & Energy Systems* **8**, 66–74 (1986).

[7] Bolognani, S. & Zampieri, S. On the existence and linear approximation of the power flow solution in power distribution networks. *IEEE Transactions on Power Systems* **31**, 163–172 (2015).

[8] Chiang, H. & Baran, M. On the existence and uniqueness of load flow solution for radial distribution power networks. *IEEE Transactions on Circuits and Systems* **37**, 410–416 (1990).

[9] Lesieutre, B. C., Sauer, P. W. & Pai, M. A. Existence of solutions for the network/load equations in power systems. *IEEE Transactions on Circuits and Systems I: Fundamental Theory and Applications* **46**, 1003–1011 (1999).

[10] Dib, W., Barabanov, A. E., Ortega, R. & Lamnabhi-Lagarrigue, F. An explicit solution of the power balance equations of structure preserving power system models. *IEEE Transactions on Power Systems* **24**, 759–765 (2009).

[11] Sanchez, S., Ortega, R., Bergna, G., Molinas, M. & Grino, R. Conditions for existence of equilibrium points of systems with constant power loads. In *IEEE Conf. on Decision and Control*, 3641–3646 (Florence, Italy, 2013).

[12] Grijalva, S. & Sauer, P. W. A necessary condition for power flow Jacobian singularity based on branch complex flows. *IEEE Transactions on Circuits and Systems I: Fundamental Theory and Applications* **52**, 1406–1413 (2005).

[13] Grijalva, S. Individual branch and path necessary conditions for saddle-node bifurcation voltage collapse. *IEEE Transactions on Power Systems* **27**, 12–19 (2012).

[14] Kundur, P. *Power System Stability and Control* (McGraw-Hill, 1994).

[15] Skar, S. J. Stability of multi-machine power systems with nontrivial transfer conductances. *SIAM Journal on Applied Mathematics* **39**, 475–491 (1980).

[16] Baillieul, J. & Byrnes, C. I. Remarks on the number of solutions to the load flow equations for a power system with electrical losses. In *IEEE Conf. on Decision and Control*, vol. 21, 919–924 (Orlando, FL, USA, 1982).

[17] Horn, R. A. & Johnson, C. R. *Matrix Analysis* (Cambridge University Press, 1985).

[18] Machowski, J., Bialek, J. W. & Bumby, J. R. *Power System Dynamics* (John Wiley & Sons, 2008), 2 edn.

[19] Hiskens, I. A. & Chakrabarti, B. B. Direct calculation of reactive power limit points. *International Journal of Electrical Power & Energy Systems* **18**, 121 – 129 (1996).

[20] Hiskens, I. A. Analysis tools for power systems — contending with nonlinearities. *Proceedings of the IEEE* **83**, 1573–1587 (2002).

[21] Simpson-Porco, J. W., Dörfler, F. & Bullo, F. Voltage stabilization in microgrids via quadratic droop control. In *IEEE Conf. on Decision and Control*, 7582–7589 (Florence, Italy, 2013).

[22] Gentile, B., Simpson-Porco, J. W., Dörfler, F., Zampieri, S. & Bullo, F. On reactive power flow and voltage stability in microgrids. In *American Control Conference*, 759–764 (Portland, OR, USA, 2014).

[23] Taylor, C. W. *Power System Voltage Stability* (McGraw-Hill, 1994).

[24] Pal, M. K. Voltage stability: analysis needs, modelling requirement, and modelling adequacy. *Generation, Transmission and Distribution, IEE Proceedings C* **140**, 279–286 (1993).

[25] Dobson, I. The irrelevance of load dynamics for the loading margin to voltage collapse and its sensitivities. In *Bulk Power System Voltage Phenomena - III* (Davos, Switzerland, 1994).

[26] Dörfler, F. & Bullo, F. Novel insights into lossless AC and DC power flow. In *IEEE Power & Energy Society General Meeting* (Vancouver, BC, Canada, 2013).

[27] Dörfler, F., Chertkov, M. & Bullo, F. Synchronization in complex oscillator networks and smart grids. *Proceedings of the National Academy of Sciences* **110**, 2005–2010 (2013).

[28] Pal, M. K. Voltage stability conditions considering load characteristics. *IEEE Transactions on Power Systems* **7**, 243–249 (1992).

[29] Milanovic, J. V., Yamashita, K., Villanueva, S. M., Djokic, S. Z. & Korunovic, L. M. International industry practice on power system load modeling. *IEEE Transactions on Power Systems* **28**, 3038–3046 (2013).

[30] Ilić, M. Network theoretic conditions for existence and uniqueness of steady state solutions to electric power circuits. In *IEEE International Symposium on Circuits and Systems*, 2821–2828 (San Diego, CA, USA, 1992).

[31] Sauer, P. W. & Pai, M. A. Power system steady-state stability and the load-flow Jacobian. *IEEE Transactions on Power Systems* **5**, 1374–1383 (1990).

[32] Overbye, T. J. Effects of load modelling on analysis of power system voltage stability. *International Journal of Electrical Power & Energy Systems* **16**, 329–338 (1994).

[33] Van Cutsem, T. & Vournas, C. *Voltage Stability of Electric Power Systems* (Springer, 1998).

[34] Venikov, V. A., Stroev, V. A., Idelchick, V. I. & Tarasov, V. I. Estimation of electrical power system steady-state stability in load flow calculations. *IEEE Transactions on Power Apparatus and Systems* **94**, 1034–1041 (1975).

[35] Löf, P.-A., Hill, D. J., Arnborg, S. & Andersson, G. On the analysis of long-term voltage stability. *International Journal of Electrical Power & Energy Systems* **15**, 229–237 (1993).

[36] Van Cutsem, T. Voltage instability: phenomena, countermeasures, and analysis methods. *Proceedings of the IEEE* **88**, 208–227 (2000).

[37] Meyer, C. D. *Matrix Analysis and Applied Linear Algebra* (SIAM, 2001).

[38] Rudin, W. *Principles of Mathematical Analysis*. International Series in Pure and Applied Mathematics (McGraw-Hill, 1976), 3 edn.

[39] Overbye, T. J., Dobson, I. & DeMarco, C. L. Q-V curve interpretations of energy measures for voltage security. *IEEE Transactions on Power Systems* **9**, 331–340 (1994).

[40] Chiang, H.-D. & Jean-Jumeau, R. Toward a practical performance index for predicting voltage collapse in electric power systems. *IEEE Transactions on Power Systems* **10**, 584–592 (1995).

[41] Abe, S. & Isono, A. Determination of power system voltage stability. Part 1: Theory. *Electrical Engineering in Japan* **96**, 171–178 (1976).

[42] Reis, C. & Barbosa, F. P. M. A comparison of voltage stability indices. In *IEEE Mediterranean Electrotechnical Conference*, 1007–1010 (Malaga, Spain, 2006).

[43] Carlson, D. & Schneider, H. Inertia theorems for matrices: The semidefinite case. *Journal of Mathematical Analysis and Applications* **6**, 430–446 (1963).

[44] Dobson, I. & Lu, L. Computing an optimum direction in control space to avoid stable node bifurcation and voltage collapse in electric power systems. *IEEE Transactions on Automatic Control* **37**, 1616–1620 (1992).

[45] Cañizares, C. A. Calculating optimal system parameters to maximize the distance to saddle-node bifurcations. *IEEE Transactions on Circuits and Systems I: Fundamental Theory and Applications* **45**, 225–237 (1998).

[46] Capitanescu, F. & Van Cutsem, T. Unified sensitivity analysis of unstable or low voltages caused by load increases or contingencies. *IEEE Transactions on Power Systems* **20**, 321–329 (2005).

[47] Cutsem, T. V. A method to compute reactive power margins with respect to voltage collapse. *IEEE Transactions on Power Systems* **6**, 145–156 (1991).

[48] Hiskens, I. A. & Davy, R. J. Exploring the power flow solution space boundary. *IEEE Transactions on Power Systems* **16**, 389–395 (2001).

[49] Dörfler, F. & Bullo, F. Kron reduction of graphs with applications to electrical networks. *IEEE Transactions on Circuits and Systems I: Regular Papers* **60**, 150–163 (2013).

[50] Horn, R. A. & Johnson, C. R. *Topics in Matrix Analysis* (Cambridge University Press, 1994).

[51] Zimmerman, R. D., Murillo-Sánchez, C. E. & Thomas, R. J. MATPOWER: Steady-state operations, planning, and analysis tools for power systems research and education. *IEEE Transactions on Power Systems* **26**, 12–19 (2011).

[52] Grigg, C. *et al.* The IEEE Reliability Test System - 1996. A report prepared by the Reliability Test System Task Force of the Application of Probability Methods Subcommittee. *IEEE Transactions on Power Systems* **14**, 1010–1020 (1999).
